# Predictive performance of interferon-gamma release assays and the tuberculin skin test for incident tuberculosis: an individual participant data meta-analysis

**DOI:** 10.1016/j.eclinm.2022.101815

**Published:** 2023-01-05

**Authors:** Yohhei Hamada, Rishi K. Gupta, Matteo Quartagno, Abbie Izzard, Carlos Acuna-Villaorduna, Neus Altet, Roland Diel, Jose Dominguez, Sian Floyd, Amita Gupta, Helena Huerga, Edward C. Jones-López, Aarti Kinikar, Christoph Lange, Frank van Leth, Qiao Liu, Wei Lu, Peng Lu, Irene Latorre Rueda, Leonardo Martinez, Stanley Kimbung Mbandi, Laura Muñoz, Elisabeth Sánchez Padilla, Mandar Paradkar, Thomas Scriba, Martina Sester, Kwame Shanaube, Surendra K. Sharma, Rosa Sloot, Giovanni Sotgiu, Kannan Thiruvengadam, Richa Vashishtha, Ibrahim Abubakar, Molebogeng X. Rangaka

**Affiliations:** a Institute for Global Health, University College London, London, United Kingdom; b MRC Clinical Trials Unit, Institute of Clinical Trials and Methodology, University College London, London, United Kingdom; c Section of Infectious Diseases, Boston University Medical Center, Boston, MA, USA; d Unitat de Tuberculosis, Hospital Universitari Vall d’Hebron-Drassanes, Barcelona, Spain; e Unitat de TDO de la Tuberculosis ‘Servicios Clínicos’, Barcelona, Spain; f Institute for Epidemiology, University Hospital Schleswig-Holstein, Campus Kiel, Kiel, Germany; g Institut d'Investigació Germans Trias i Pujol, CIBER Enfermedades Respiratorias, Instituto de Salud Carlos III, Universitat Autònoma de Barcelona, Barcelona, Spain; h Department of Infectious Disease Epidemiology, London School of Hygiene & Tropical Medicine, London, United Kingdom; i Johns Hopkins University School of Medicine, Baltimore, MD, USA; j Epicentre, Paris, France; k Division of Infectious Diseases, Department of Medicine, Keck School of Medicine of USC, University of Southern California, Los Angeles, CA, USA; l Byramjee Jeejeebhoy Government Medical College and Sassoon General Hospital, Pune, Maharashtra, India; m Division of Clinical Infectious Diseases, Research Center Borstel, Borstel, Germany; n German Center for Infection Research (DZIF), Clinical Tuberculosis Unit, Borstel, Germany; o Respiratory Medicine & International Health, University of Lübeck, Lübeck, Germany; p Baylor College of Medicine and Texas Children's Hospital, Houston, TX, USA; q Tuberculosis Network European Trials Group (TBnet), Borstel, Germany; r Department of Health Sciences, VU University, Amsterdam, the Netherlands; s Amsterdam Public Health research institute, Amsterdam, the Netherlands; t Department of Chronic Communicable Disease, Center for Disease Control and Prevention of Jiangsu Province, Nanjing, Jiangsu Province, PR China; u Department of Epidemiology, School of Public Health, Boston University, Boston, MA, USA; v South African Tuberculosis Vaccine Initiative, Institute of Infectious Disease and Molecular Medicine, and Division of Immunology, Department of Pathology, University of Cape Town, South Africa, Western Cape, South Africa; w Department of Clinical Sciences, University of Barcelona, Barcelona, Spain; x Byramjee Jeejeebhoy Government Medical College-Johns Hopkins University Clinical Research Site, Pune, Maharashtra, India; y Johns Hopkins India, Pune, Maharashtra, India; z Department of Transplant and Infection Immunology, Saarland University, Homburg, Germany; aa Zambart, Lusaka, Zambia; ab Department of Internal Medicine, All India Institute of Medical Sciences, New Delhi, India; ac Department of Molecular Medicine, Jamia Hamdard Institute of Molecular Medicine, Hamdard University, Delhi, India; ad Departments of General Medicine & Pulmonary Medicine, JNMC, Datta Meghe Institute of Medical Sciences, Maharashtra, India; ae Desmond Tutu TB Centre, Department of Paediatrics and Child Health, Faculty of Medicine and Health Sciences, Stellenbosch University, Cape Town, South Africa; af Clinical Epidemiology and Medical Statistics Unit, Department of Medicine, Surgery and Pharmacy, University of Sassari, Sassari, Italy; ag National Institute for Research in Tuberculosis, Indian Council of Medical Research, Chennai, Tamil Nadu, India; ah School of Public Health, and Clinical Infectious Disease Research Institute-Africa, University of Cape Town, Cape Town, South Africa

**Keywords:** LTBI, IGRA, Tuberculin skin test, Prophylaxis, Prevention

## Abstract

**Background:**

Evidence on the comparative performance of purified protein derivative tuberculin skin tests (TST) and interferon-gamma release assays (IGRA) for predicting incident active tuberculosis (TB) remains conflicting. We conducted an individual participant data meta-analysis to directly compare the predictive performance for incident TB disease between TST and IGRA to inform policy.

**Methods:**

We searched Medline and Embase from 1 January 2002 to 4 September 2020, and studies that were included in previous systematic reviews. We included prospective longitudinal studies in which participants received both TST and IGRA and estimated performance as hazard ratios (HR) for the development of all diagnoses of TB in participants with dichotomised positive test results compared to negative results, using different thresholds of positivity for TST. Secondary analyses included an evaluation of the impact of background TB incidence. We also estimated the sensitivity and specificity for predicting TB. We explored heterogeneity through pre-defined sub-group analyses (e.g. country-level TB incidence). Publication bias was assessed using funnel plots and Egger's test. This review is registered with PROSPERO, CRD42020205667.

**Findings:**

We obtained data from 13 studies out of 40 that were considered eligible (N = 32,034 participants: 36% from countries with TB incidence rate ≥100 per 100,000 population). All reported data on TST and QuantiFERON Gold in-Tube (QFT-GIT). The point estimate for the TST was highest with higher cut-offs for positivity and particularly when stratified by bacillus Calmette–Guérin vaccine (BCG) status (15 mm if BCG vaccinated and 5 mm if not [TST^5/15 mm^]) at 2.88 (95% CI 1.69–4.90). The pooled HR for QFT-GIT was higher than for TST at 4.15 (95% CI 1.97–8.75). The difference was large in countries with TB incidence rate <100 per 100,000 population (HR 10.38, 95% CI 4.17–25.87 for QFT-GIT VS. HR 5.36, 95% CI 3.82–7.51 for TST^5/15 mm^) but much of this difference was driven by a single study (HR 5.13, 95% CI 3.58–7.35 for TST^5/15 mm^ VS. 7.18, 95% CI 4.48–11.51 for QFT-GIT, when excluding the study, in which all 19 TB cases had positive QFT-GIT results). The comparative performance was similar in the higher burden countries (HR 1.61, 95% CI 1.23–2.10 for QFT-GIT VS. HR 1.72, 95% CI 0.98–3.01 for TST^5/15 mm^). The predictive performance of both tests was higher in countries with TB incidence rate <100 per 100,000 population. In the lower TB incidence countries, the specificity of TST (76% for TST^5/15 mm^) and QFT-GIT (74%) for predicting active TB approached the minimum World Health Organization target (≥75%), but the sensitivity was below the target of ≥75% (63% for TST^5/15 mm^ and 65% for QFT-GIT). The absolute differences in positive and negative predictive values between TST^15 mm^ and QFT-GIT were small (positive predictive values 2.74% VS. 2.46%; negative predictive values 99.42% VS. 99.52% in low-incidence countries). Egger's test did not show evidence of publication bias (0.74 for TST^15 mm^ and p = 0.68 for QFT-GIT).

**Interpretation:**

IGRA appears to have higher predictive performance than the TST in low TB incidence countries, but the difference was driven by a single study. Any advantage in clinical performance may be small, given the numerically similar positive and negative predictive values. Both IGRA and TST had lower performance in countries with high TB incidence. Test choice should be contextual and made considering operational and likely clinical impact of test results.

**Funding:**

YH, IA, and MXR were supported by the 10.13039/501100000272National Institute for Health and Care Research (NIHR), United Kingdom (RP-PG-0217-20009). MQ was supported by the 10.13039/501100000265Medical Research Council [MC_UU_00004/07].

**Research in context**

**Evidence before this study**

We searched Medline and Embase from 1 January 2002 to 4 September 2020 with search terms related to ‘tuberculosis (TB)’, ‘ interferon-gamma release assays (IGRA)’, and ‘tuberculin skin test (TST)’ and studies that were included in previous systematic reviews. Current global policy strongly recommends, albeit with very low certainty of evidence, that either TST or IGRA can be used for TB infection screening in all populations and settings regardless of the background TB incidence. This was based on a previous meta-analysis of head-to-head studies which showed both tests imprecisely indicated an increased risk of incident TB, and although IGRA had higher relative risk estimates than the TST, confidence intervals overlapped. The higher relative risk estimates of IGRA was driven by studies with a high risk of incorporation where these were conducted in low-incidence resource-rich countries that had widely adopted the test and used positive IGRA results to trigger investigations of active TB only in test positives. The conclusion was that, in head-to-head evaluations, IGRA showed no clear predictive advantage over the TST and that the tests had similar predictive performance. In contrast, a recent systematic review concluded that IGRA had better predictive performance than TST. However, the primary analysis was indirect and not restricted to head-to-head studies. Both these previous reviews conducted study-level meta-analyses. This approach is prone to bias due to systematic differences between study settings, participants, and follow-up intervals. The comparative prognostic ability of TST and IGRA has therefore remained unclear, due to these conflicting analyses. We updated the search on 11 December 2022 with the same search terms, which identified two prospective cohort studies in low TB incidence countries that tested participants with both TST and IGRA; however, one (Calzada-Hernández 2022) included a small sample size and event rate (two TB cases in 283 participants), and the other (Ho 2022) reported that data on TB incidence were not yet finalised at the time of manuscript writing.

**Added value of this study**

To inform policy, we conducted an individual participant data meta-analysis to compare the predictive performance of TST and IGRA. This enabled the exploration of systematic differences between study settings, study designs, TST cut-off values, participants and follow-up intervals and address limitations of previous systematic reviews. We found that QuantiFERON Gold in-Tube (QFT-GIT) had a higher point estimate of predictive performance. The difference appears to be driven by one study, in which all TB cases had positive QFT-GIT results in a low TB incidence setting. An analysis without this study resulted in similar point estimates for TST and IGRA, suggesting comparable performance. When we stratified study countries into two groups based on their TB incidence rate, the pooled predictive performance for TST and IGRA was higher in countries with TB incidence rates <100 per 100,000 population. In low TB incidence settings, the higher predictive performance of IGRA resulted in a minimal difference in positive and negative predictive values for incident TB. The performance between TST and IGRA was similar in high TB incidence countries.

In low TB incidence countries, both IGRA and TST had around 75% specificity (74% for QFT-GIT and 76% for TST using 15 mm cut-off for bacillus Calmette−Guérin vaccinated and 5 mm for unvaccinated) over two years, which is at World Health Organization (WHO) targets in low TB incidence countries (>75%) but the sensitivity was below the target. The performance of both tests was worse in countries with higher TB incidence and did not meet WHO targets.

**Implications of all the available evidence**

IGRA appears to predict TB incidence better than TST in low TB incidence settings, but the difference was largely driven by a single study, and the absolute predictive advantage may be small. By contrast, the tests perform similarly in high TB incidence countries. Both have limited prognostic ability, particularly in settings with higher TB incidence. While we await development, validation and roll-out of new tests with improved prognostic ability, other strategies such as the use of quantitative TB infection results combined with demographic and clinical factors in multivariable algorithms could be explored in high TB incidence countries.

## Introduction

The World Health Organization (WHO) recommends preventive treatment in populations who are at risk for the development of tuberculosis (TB).^1^ Individuals who are infected with *Mycobacterium tuberculosis* without clinical manifestation, known as latent TB infection, are at risk for the development of TB.^1^ Two types of indirect diagnostics are recommended to detect individuals who might benefit from TB preventive treatment: purified protein derivative (PPD) tuberculin skin test (TST) and interferon-gamma release assays (IGRA).^1^ In both, a simple dichotomization to positive and negative denotes probable infection or not, respectively. None are considered “gold standard tests for TB infection” since they measure immune sensitization to mycobacterial antigens and, consequently, they cannot differentiate current TB infection from past infection, as well as infection from disease.^2^ TST also lacks specificity for TB infection owing to cross-reactivity among individuals vaccinated with *Mycobacterium bovis* bacillus Calmette−Guérin vaccines (BCG) or infected by nontuberculous mycobacteria (NTM). IGRA, including T-SPOT.TB and QuantiFERON-TB Gold in tube (QFT-GIT) or Plus (QFT-Plus), use *M. tuberculosis*-specific antigens, ESAT-6 and CFP-10. Thus, they are not affected by BCG or most NTM (other than *Mycobacterium kansasii, marinum and szulgai*) and are expected to increase the diagnostic specificity.

In head-to-head comparisons,^3^^,^^4^ previous meta-analyses using aggregated data suggest IGRA's increased specificity does not appear to confer greater prognostic ability to predict individuals who will progress from infection to disease than the TST. Despite IGRA's higher relative risk estimates for incident TB, confidence intervals were imprecise and overlapped. In addition, the higher relative risk estimates of IGRA was driven by studies with a high risk of incorporation where these were conducted in low-incidence resource-rich countries that had widely adopted the test and used positive IGRA results to trigger investigations of active TB only in test positives.^4^ These findings underpinned the initial policy on the use of IGRA for prediction of TB in low TB incidence countries, and later updates were used to inform global policy that recommends the use of either test interchangeably in all settings regardless of the background TB incidence.

Most recently, Zhou et al. conducted an updated meta-analysis, which reported superior predictive performance of IGRA over TST.^5^ However, the primary analysis was not restricted to head-to-head analysis, where participants received both tests. Thus, the results could be affected by the differences in the set of studies included for each test. In addition, without individual participant data, systematic differences between study settings, study designs, TST cut-off values, participants and follow-up intervals cannot be fully considered in meta-analyses using aggregate data. The definitive evaluation of whether IGRA improves the predictive performance over TST using a robust methodology is essential for policymakers to decide which test to roll out. This is especially important for low and middle-income countries, which are now expanding TB preventive treatment to all household contacts, for whom testing for TB infection are desirable in light of the latest guidelines.^1^ However, the previous review by Zhou et al. included limited data from those countries.^6^

We conducted a systematic review and individual participant data (IPD) meta-analysis to directly compare the predictive ability of the TST and IGRAs in both high and low TB incidence countries. First, as recommended by the WHO framework for evaluation of tests for TB infection, we estimated the risk for the development of active TB in participants with positive test results compared to negative results for both TST and IGRA.^7^ Second, we compared their predictive ability when using quantitative test results. Lastly, we investigated how the performance differed by setting and population to explore the best test for specific risk groups. We further evaluated the predictive performance against the minimum targets set by WHO (>75% sensitivity and specificity over a two-year interval).^8^

## Methods

We performed a systematic review and IPD meta-analysis following Preferred Reporting Items for a Systematic Review and Meta-analysis of Individual Participant Data (PRISMA-IPD) guidance.^9^ The protocol for this review is registered on PROSPERO (https://www.crd.york.ac.uk/prospero/display_record.php?RecordID=205667) This study involved analyses of anonymised data from previously published cohort studies, with data pooling via a secure system. Ethical approvals for sharing of data were sought and obtained by contributors of individual participant data, where required. Data were stored in a data safe haven held by the University College London.

### Eligibility criteria

We adapted the eligibility criteria from our previous systematic review that developed a multivariable model to predict the development of active TB, limited to low TB incidence countries (annual incidence ≤20/100,000 persons; PROSPERO CRD42018115357). The previous review included TST and IGRA results as model parameters but did not compare their predictive performance. For the present review, we included studies from low to high TB incidence settings which systematically tested participants with both TST and at least one commercially available IGRA (paired) and followed them for incident TB for the median follow-up duration of at least one year in any world region (Table S1 in the appendix).

### Definitions

For the primary analysis, incident TB disease was defined as bacteriologically confirmed or clinically diagnosed TB as per study definitions. In the sensitivity analysis, we restricted to bacteriologically confirmed TB. We excluded cases of TB disease diagnosed within 42 days of enrolment, which were deemed cases of prevalent TB in line with the definition used previously.^10^ We explored alternative definitions of prevalent TB in sensitivity analyses (see below).

For TST, we explored multiple cut-off values: ≥5 mm (TST^5 mm^), ≥10 mm (TST^10 mm^), ≥15 mm (TST^15 mm^), and BCG-stratified (TST^5/15 mm^), which is ≥15 mm for BCG-vaccinees and ≥5 mm for unvaccinated. The results of QFT-GIT were defined as per its package insert.^11^ For T-SPOT.TB results, we classified “borderline positive” and “borderline negative” results as positive and negative, respectively, as previously.^8^ No studies reported data on QFT-plus. Indeterminate results were excluded for both QFT-GIT and TSPOT.TB.

### Search strategy

We identified relevant studies by updating the search conducted by Gupta et al., which included studies published in low TB incidence settings since 1 January 2002 until December 2018.^10^ We ran the updated search using Medline and Embase to identify papers published since 1 January 2019. The search strategy included related terms for ‘TB’, ‘IGRA’, and ‘TST’ for each search engine. The full search strategy for Medline is shown in Appendix 2. The search was not restricted by language. We supplemented the search by consulting experts in the field. Additionally, we screened all studies that were retained for full-text review by Gupta et al., which searched for longitudinal studies assessing the risk of progression to TB in individuals tested for TB infection.^10^ While the review and analysis by Gupta et al. focused on studies conducted in low TB incidence settings, all relevant studies, regardless of settings, were retained for the full-text review. We also reviewed studies included in previous reviews, including those that informed WHO guidelines.^3^^,^^5^^,^^12^

### Study selection, data collection, and quality assessment

Two investigators (YH and RG) independently reviewed titles and abstracts identified through the electronic search. Two investigators (YH and AI) screened full-text of relevant articles in duplicate. The two reviewers discussed disagreements, and if consensus was not reached, they were resolved through arbitration by a third reviewer (RG or MXR).

We contacted authors of eligible studies by email to invite them to contribute to anonymised IPD, including a set of pre-specified variables (Appendix 3). Where there is no response, we followed up with them multiple times. We checked IPD for consistency with data reported in study publications and potentially invalid and implausible values. Issues raised were resolved by contacting the study authors. We treated height, weight, and body mass index (BMI) which are biologically implausible as missing, following criteria used in previous studies.^12^^,^^13^ IPD were mapped to a master variables list, and variables were standardised across IPD for synthesis. Two investigators (YH and AI) independently conducted a quality assessment of included studies using a modified version of the Newcastle–Ottawa Scale (NOS) (Appendix 4). Comparability was considered irrelevant and thus was not assessed since participants received both tests, and TB risk was compared between test-positive and-negative individuals; hence two groups were inherently different.

### Statistical analysis

#### Handling of missing data

We excluded participants with missing follow-up durations. Most (95%) of them lacked outcome data and contributed little information. We assumed Missing at Random (MAR) given all the available information was the most likely missingness mechanism for the remaining observations; hence, we conducted multiple imputations using multilevel fully conditional specification accounting for clustering by study. The models included the outcome (TB disease), test results (TST, QFT-GIT, and TSPOT), variables used for sub-group analysis (age, TB disease incidence in study countries, contact history, human immunodeficiency virus infection (HIV) status, and body mass index (BMI)) and auxiliary variables, including previous BCG vaccination, previous TB, smoking, preventive TB treatment, and interaction terms (See Appendix 1 for details).

#### Predictive performance of binary test results

We intended to synthesise all the available evidence, so we collected all the available data rather than performing formal sample size calculations.

Our primary effect measures were hazard ratios (HR) for the development of TB disease in participants with positive test results compared with those with negative results, for each index test. The use of HR, rather than diagnostic accuracy measures of sensitivity and specificity, allowed accounting for person-time. We conducted a one-stage meta-analysis to estimate pooled hazard ratios in our primary analysis using mixed effects Cox regression model. The model included study-specific intercepts and random slopes for test results by study. The meta-analysis was limited to participants who did not receive TB preventive treatment. We compared pooled estimates across head-to-head studies by examining point estimates and 95% confidence intervals (CIs). We investigated heterogeneity by visualizing forest plots, and we estimated the proportion of variation across studies attributable to between-study heterogeneity (I^2^ statistics, by fitting a two-stage model). Additionally, to explore the source of heterogeneity, we assessed changes in predictive performance by setting and population characteristics.

The analysis produces HR for each test and does not give a single value indicating the relative performance of one test VS. the other. Thus, we adopted a method used in Abubakar et al.^13^ to make pair-wise comparisons of the predictive performance of TST with different cut-off values VS. QFT-GIT or T-SPOT.TB. Accordingly, we fitted a mixed effect logistic regression model with a positive result as an outcome and an interaction term between test type and the development of TB disease to assess the strength of the association between positive results and TB disease development and how it differs by test type. The model accounted for clustering within individuals and studies. The results from this model were expressed in the ratios of two odds ratios for TST and IGRA, respectively, for positive test results in participants who developed TB disease compared with those who did not.

We planned to conduct the same analysis in those who received TB preventive treatment; however, the small number of participants given treatment (n = 1838) and subsequent disease events precluded this analysis.

#### Predictive performance of quantitative test results

We also compared the predictive ability of TST and IGRA using their quantitative results to maximise the use of all the available information rather than dichotomizing them, as previously.^10^ We transformed the quantitative values to a percentile scale using the study data set (see Appendix 5 for a look-up table). The transformation allowed the comparison of the test results reported using different scales. Then, we examined the association between normalised results for each test and the risk of incident TB disease. We performed mixed-effects Cox proportional hazards models with restricted cubic splines with three knots for quantitative test results at recommended intervals.^14^ We fitted a one-stage model with study-specific intercepts and random slopes for test results by study.

#### Predictive performance by population and setting and sensitivity analysis

Where possible, we presented sub-group analysis and assessed within-study interactions (see Appendix 1).

We estimated the sensitivity and specificity of tests to predict TB disease incidence over two years by setting and compared them against the minimum performance targets of >75% sensitivity and specificity set by WHO (Appendix 1).^8^ To explore the clinical impact, we presented positive and negative predictive values (PPV and NPV, respectively) in hypothetical populations with different pre-test probabilities of developing TB disease.

We repeated the analyses: (1) using a two-stage meta-analysis fitting Poisson regression models to estimate incident rate ratios for TB; (2) using shorter (<14 days) and longer (<180 days) temporal definitions of prevalent TB disease; and (3) including only incident TB disease (<42 days) events with microbiological confirmation. Furthermore, we repeated sub-group analysis by TB incidence by excluding HIV-positive individuals to assess the impact of confounding by HIV status.

In order to assess the impact of studies that did not provide IPD, we combined their aggregated data with those from studies with IPD and conducted meta-analysis. We used data among participants not given TB preventive treatment and estimated pooled odds ratio using mixed effects logistic regression models (See Appendix 1).

We assessed publication bias using funnel plots and Egger's test.

### Role of the funding source

The funder of the study had no role in study design, data collection, data analysis, data interpretation, or writing of the report.

## Results

### Characteristics of included studies

**Fig. 1:**
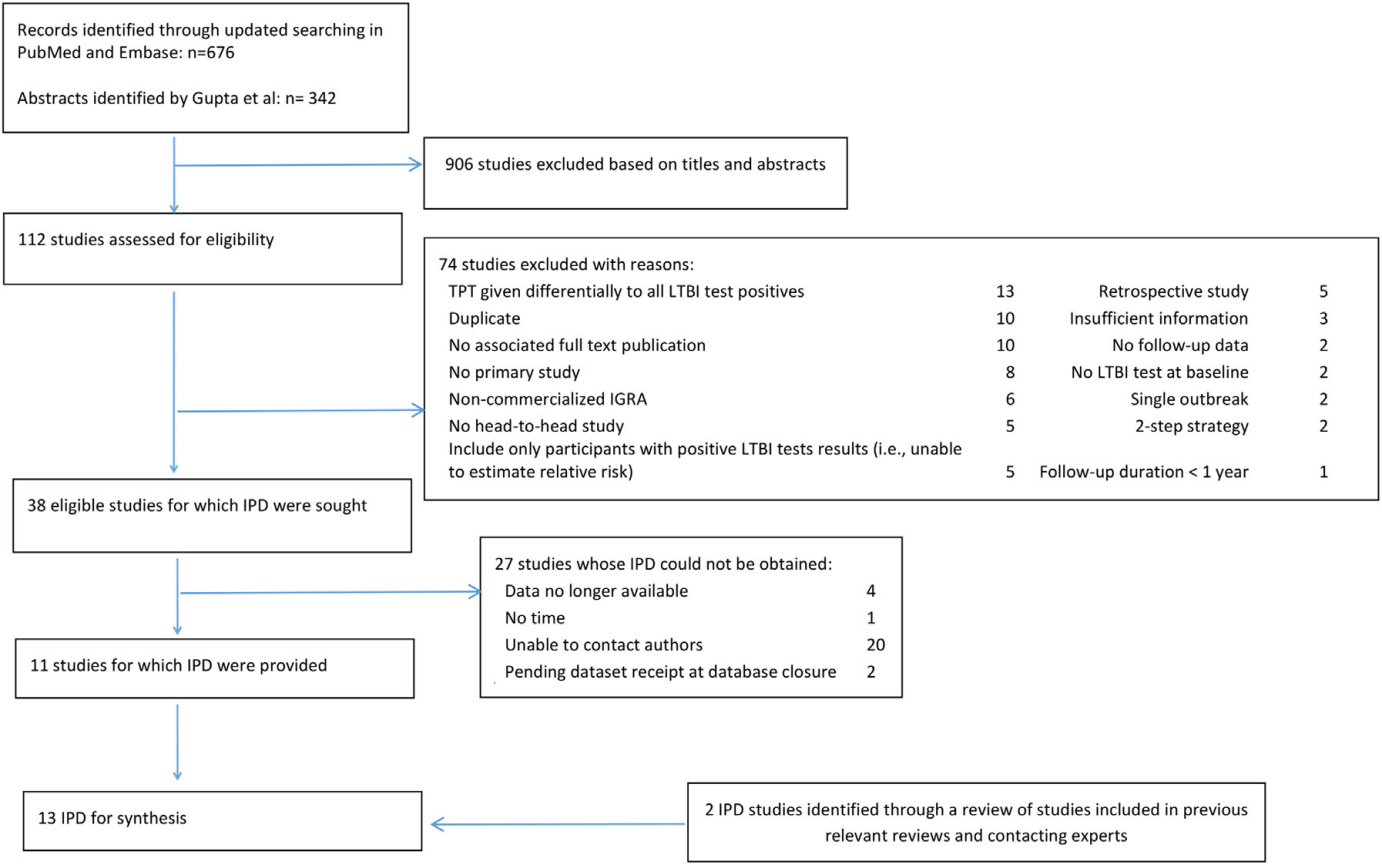
**Study selection.** IPD: individual participant data; TPT: TB preventive treatment; LTBI; latent tuberculosis infection.

From 1018 titles and abstracts screened, we identified 38 eligible studies, for which IPD were sought. IPD were obtained from 11 studies^13^^,^^15^, ^16^, ^17^, ^18^, ^19^, ^20^, ^21^, ^22^, ^23^, ^24^ while two additional datasets were obtained through other sources and included (Fig. S1).^25^^,^^26^ IPD could not be obtained from 27 studies^27^, ^28^, ^29^, ^30^, ^31^, ^32^, ^33^, ^34^, ^35^, ^36^, ^37^, ^38^, ^39^, ^40^, ^41^, ^42^, ^43^, ^44^, ^45^, ^46^, ^47^, ^48^, ^49^, ^50^, ^51^, ^52^, ^53^ (n = 20,333) most commonly because of non-response (n = 20) (Fig. 1). Characteristics of those studies are presented in Table S2. Most of them (24/27) were conducted in countries with TB incidence rate <100 per 100,000 population, mainly including adults.

The 13 included study datasets comprised 33,093 participants so that while only 33% of studies were included, these included 62% of the potential participants. Among these, we excluded 141 participants who had prevalent TB disease, 917 participants with missing follow-up durations, and one with negative follow-up duration, leaving 32,034 participants for subsequent analysis. Among 917 participants with missing follow-up duration, 871 (95.0%) had missing data on the incidence of TB disease, which provides little information about the predictive performance of TST VS. IGRA.

The median (IQR) follow-up duration was 3.6 (2.0–5.6) years; 518 (1.6%) participants developed incident TB disease (Table 1). The median (IQR) time to the development of TB disease was 1.1 years (0.5–1.7). The median time to the development of TB was slightly longer in studies where TB incidence rate was ≥100 per 100,000 than that in the other studies (1.20 VS. 0.90 years). Among participants, 39%, 8.9%, and 14.8% were screened for TB infection because of contact with TB, immunosuppression, and migration from high TB incidence countries, respectively. Besides, 19% were general adolescents in South Africa^15^ and 17% were general adults in China.^17^ Among all participants, 7.8% were HIV-positive. Only 1% (n = 396) of the participants were children less than five years old. Participants from European countries accounted for 44.3% of the population, followed by African countries (South Africa and Zambia, 28.2%) (Tables S3 and S4). Data on TST and QFT-GIT were available in 27,400 participants (85.5%, 13 studies)^13^^,^^15^, ^16^, ^17^, ^18^, ^19^, ^20^, ^21^, ^22^, ^23^, ^24^, ^25^, ^26^ and TST and TSPOT.TB in 8612 participants (26.9%, three studies).^13^^,^^16^^,^^18^ None of the studies blinded results of TB infection tests for diagnosing incident TB.

**Table 1:** Characteristics of study participants (N = 32,034).

Variables		Missing data n (%)
Follow-up duration (median years (IQR))	3.6 years (2.0–5.6)	
Age (mean (SD))	33.05 (18.27)	68 (0.1)
Female	16,936 (52.9)	73 (0.3)
Reason for screening (%)		102 (0.3)
Contact	12,564 (39.2)	
Immunosuppression	2862 (8.9)	
Migrant	4739 (14.8)	
Others^a^	11,767 (36.7)	
Previous BCG vaccination (%)	19,372 (63.3)	5166 (16.1)
Previous TB diagnosis (%)	1855 (5.8)	9909 (30.9)
HIV positive (%)	2497 (7.8)	7559 (23.6)
BMI (mean (SD))	24·00 (5.52)	14,114 (44.1)
TB preventive treatment (%)	1883 (5.9)	859 (2.7)
Study country (%)		0 (0)
Brazil	890 (2.8)	
China	5404 (16.9)	
Europe	14,184 (44.3)	
India	2519 (7.9)	
South Africa or Zambia	9037 (28.2)	
QFT-GIT (%)		1994 (6.2)
Indeterminate	462 (1.4)	
Negative	19,210 (60.0)	
Positive	10,368 (32.4)	
TSPOT.TB (%)		17,006 (63.9)
Borderline	433 (1.4)	
Indeterminate	232 (0.7)	
Negative	7083 (22.1)	
Positive	1876 (5.9)	
TST		3205 (12.0)
≥5 mm	14,589 (45.5)	
≥10 mm	10,858 (33.9)	
≥15 mm	6124 (19.1)	

SD: standard deviation; IQR: interquartile range; BCG: bacillus Calmette−Guérin; HIV: *human immunodeficiency virus*; QFT-GIT: QuantiFERON Gold in Tube; BMI: body mass index; TST: tuberculin skin test.

a Others include HIV-negative adolescents (Mahomed 2011) and the general adults (Lu 2021).

Eleven studies achieved a NOS quality score of 7/8, and the remaining two achieved 6/8 due to unclear methods for excluding prevalent TB disease at the baseline (Table S5).

### Predictive performance of binary test results

Figure S1 presents cumulative TB incidence curves by test results for each test, which showed a higher risk of TB in participants with positive test results. The point estimates for TST predictive performance tended to be higher when a higher cut-off value was used for positivity (Table 2). The pooled HR was 2.71 (95% CI 1.63–4.50) at TST^15 mm^, 2.48 (95% CI 1.57–3.92) for TST^10 mm^, and 2.30 (95% CI 1.45–3.64) at TST^5 mm^. When TST cut-off was stratified by BCG status (i.e., TST^5/15 mm^), the HR was 2.88 (95% CI 1.69–4.90). There was great variability in effect estimates by study. For example, for TST^15 mm^, the HR ranged from 0.92 in a study in India among household contacts to 30.29 in household contacts in Spain (tau^2^ = 0.52, I^2^ = 73.1%; 95% CI 52.1–84.9%) (Fig. S2).

**Table 2:** Pooled estimates of the predictive performance of TST VS. IGRA for all TB.

Test	All studies (n = 13)		Studies with TSPOT.TB (n = 3)	
	HR^a^ (95% CI)	I^2^ (95% CI)	HR^a^ (95% CI)	I^2^ (95% CI)
TST5 mm	2.3 (1.45–3.64)	56.1 (16.2–77)	5.11 (3.16–8.25)	0 (0–89.6)
TST10 mm	2.48 (1.57–3.92)	62.2 (29.3–79.8)	5.24 (3.4–8.06)	0 (0–89.6)
TST15 mm	2.71 (1.63–4.5)	73.1 (52.1–84.9)	7.87 (3.42–18.12)	71.1 (1.8–91.5)
TST5/15 mm^b^	2.88 (1.69–4.9)	64.8 (34.8–81)	5.98 (3.51–10.21)	63 (0–89.4)
QFT-GIT	4.15 (1.97–8.75)	76.1 (58.1–86.3)	8.98 (3·17–25·41)	75.2 (18–92.5)
T-SPOT.TB	–	–	6.45 (3·32–12·55)	0 (0–89.6)

HR: hazard ratio; CI: confidence interval; QFT-GIT: QuantiFERON Gold in Tube; TST: tuberculin skin test.

When one study (Diel 2011) where HR could not be estimated directly was excluded since all TB cases had positive QFT-GIT, HR was 2.19 (95% CI 1.36–3.53) for TST5 mm, 2.49 (95% CI 1.50–4.15) for TST10 mm, 2.90 (95% CI 1.72–4.9) for TST15 mm, 2.64 (95%CI 1.52–4.59) for TST5/15 mm, and 3.09 (95% CI 1.74–5.50) for QFT-GIT.

a The risk of TB relative to participants with negative test results.

b TST5/15 mm indicates BCG-stratified cut-off, which is ≥15 mm for BCG-vaccinees and ≥5 mm for unvaccinated.

The pooled HR of QFT-GIT was 4.15 (95% CI 1.97–8.75) for incident TB, numerically higher than that for TST^5/15 mm^; however, its confidence interval was wide with a substantial overlap with that of TST^5/15 mm^ (Table 2). The two-stage meta-analysis showed similar point estimates in the predictive performance between TST^15 mm^ and QFT-GIT (HR 2.76, 95% CI 1.67–4.58 VS. HR 3.03 95% CI 1.71–5.35) (Fig. S3). This difference in the point estimates was driven by one study (n = 1414) in Germany,^19^ in which all 19 TB cases had positive QFT-GIT results; thus, HR could not be directly estimated. Without this study, the one-stage meta-analysis estimate for the HR reduced slightly to 3.12 (95% CI 1.75–5.57) for QFT-GIT, which was similar to HR 2.92 for TST^15 mm^ (95% CI 1.73–4.92). Similarly to TST, the HR of QFT-GIT varied widely from 1.31 to 46.90 (tau^2^ = 0.71, I^2^ = 76.1, 95% CI 58.1–86.3) (Fig. S2). In the study of Lu et al. conducted in China, there was the largest difference in the point estimate between QFT-GIT and TST^15 mm^ (HR 7.82; 95% CI 2.36–25.98 for QFT-GIT VS. 3.99; 95% CI 1.27–12.56 for TST^15 mm^).^17^

In the pair-wise comparisons of the test performance, the ratio of odds ratios for QFT-GIT VS. TST^15 mm^ was 1.48 (95% CI 1.03–2.13), suggesting a slightly higher predictive performance of QFT-GIT than TST^15 mm^ (Table S6). This was again driven by the same one study as above,^19^ and when it was excluded, the ratio declined, and its confidence interval crossed one (1.23; 95% CI 0.84–1.80). Likewise, the ratio for QFT-GIT VS. TST^5 mm/15 mm^ based on all studies was 1.14 (95% CI 0.72–1.81).

We compared the predictive performance of TST VS. T-SPOT.TB, using individual data from three studies with data on T-SPOT.TB.^13^^,^^16^^,^^18^ The predictive performance was similar between TST^15 mm^, TST^5/15 mm^, and T-SPOT.TB (Table 2 and Fig. S4). The pooled HRs for TST^15 mm^ and TST^5/15 mm^ were 7.87 (95% CI 3.42–18.12) and 5.98 (95% CI 3.51–10.21), respectively, compared to that of 6.45 (95% CI 3.32–12.55) for T-SPOT.TB, and 8.98 (95% CI 3.17–25.41) for QFT-GIT. Because of the small number of studies and events, the pooled estimates from the two-stage meta-analysis were very wide (Fig. S5).

The sensitivity analyses that used only bacteriologically confirmed TB and those using a shorter (within 14 days from enrolment) or longer (within 180 days) temporal definition of prevalent TB disease did not change the overall trends significantly (Tables S7 and S8).

Egger's test did not show the evidence of publication bias (Fig. S6).

### Predictive performance of quantitative test results

**Fig. 2:**
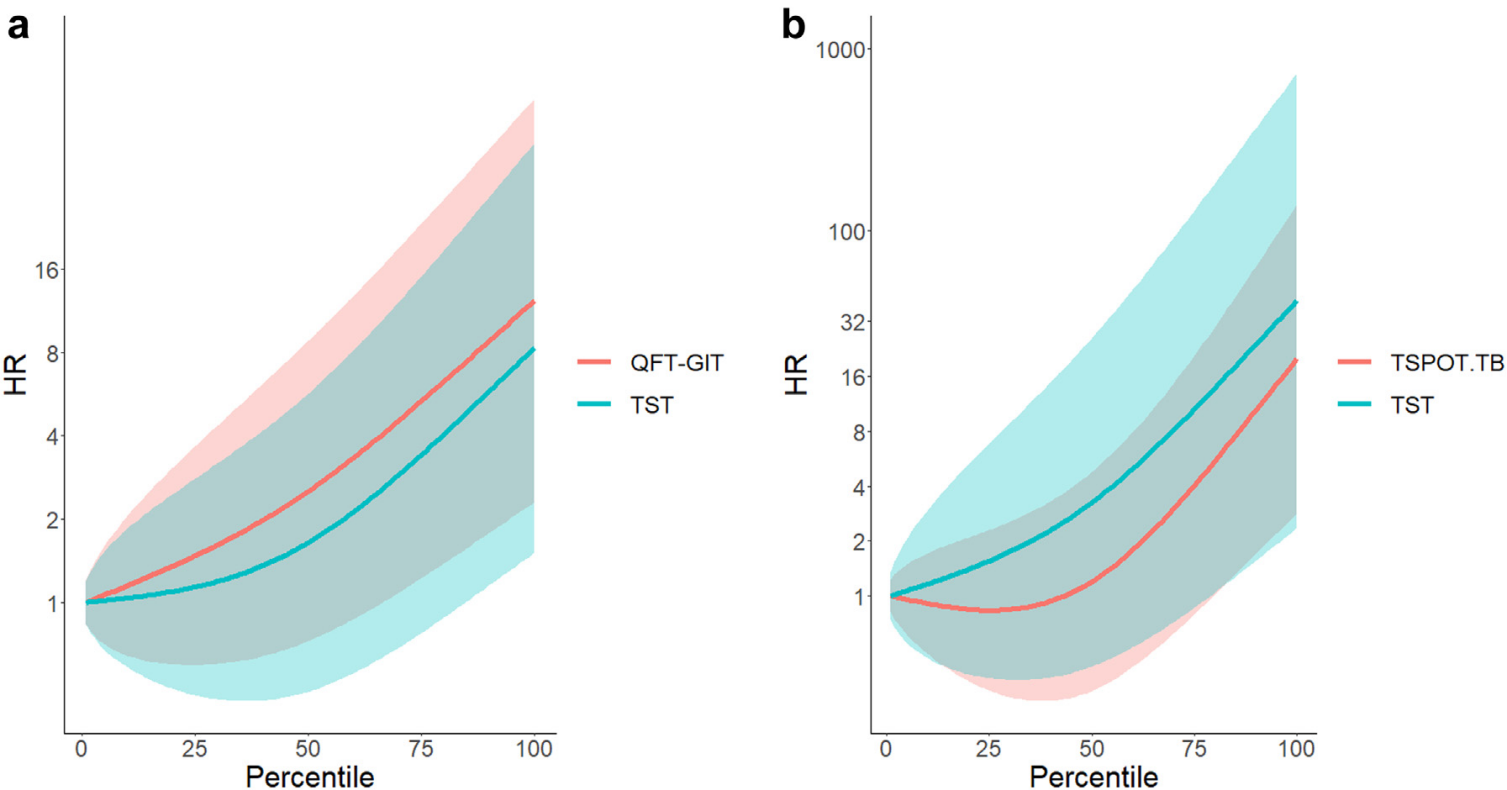
**The association between quantitative results and the risk of TB.** (a) TST and QFT-GIT. (b) TST and TSPOT-TB. TB: tuberculosis; HR: Hazard ratio; QFT-GIT: QuantiFERON TB Gold In-Tube; TST: tuberculin skin test. Quantitative results of TST, QFT-GIT, and TSPOT-TB are normalized to a percentile scale. HR indicates the risk of TB incidence with participants who have results at zero percentile scale as a reference group (i.e. HR = 1). The areas around the lines indicate 95% confidence intervals.

Fig. 2 presents changes in HR by quantitative test results relative to participants with test results at the 0th percentile (i.e., no induration for TST and 0 quantitative values for IGRA). Across all three tests, HR appeared to increase as the quantitative test results increase. For example, in participants with TST induration size at the 50th, 66th, and 83rd percentile, which corresponds to the induration sizes of 5 mm, 10 mm, and 15 mm, respectively, the HRs were 1.64 (95% CI 0.48–5.68), 2.54 (95% CI 0.63–10.29), and 4.52 (95% CI 0.95–21.36). Similarly, in participants with QFT-GIT results at the same percentile scales, which corresponds to the values of 0.09–0.099 IU/ml, 0.62–0.699 IU/ml, and 3.77–4.269 IU/ml, HRs were 2.52 (95% CI 0.72–8.78), 4.00 (95% CI 0.99–16.16), and 6.95 (95% CI 1.49–32.4). For TST VS. T-SPOT.TB, since only three studies had data, the confidence intervals were wide; no significant difference was identified, although HR for TST remained higher than T-SPOT.TB throughout the percentile scales.

### Sub-group analysis

**Fig. 3:**
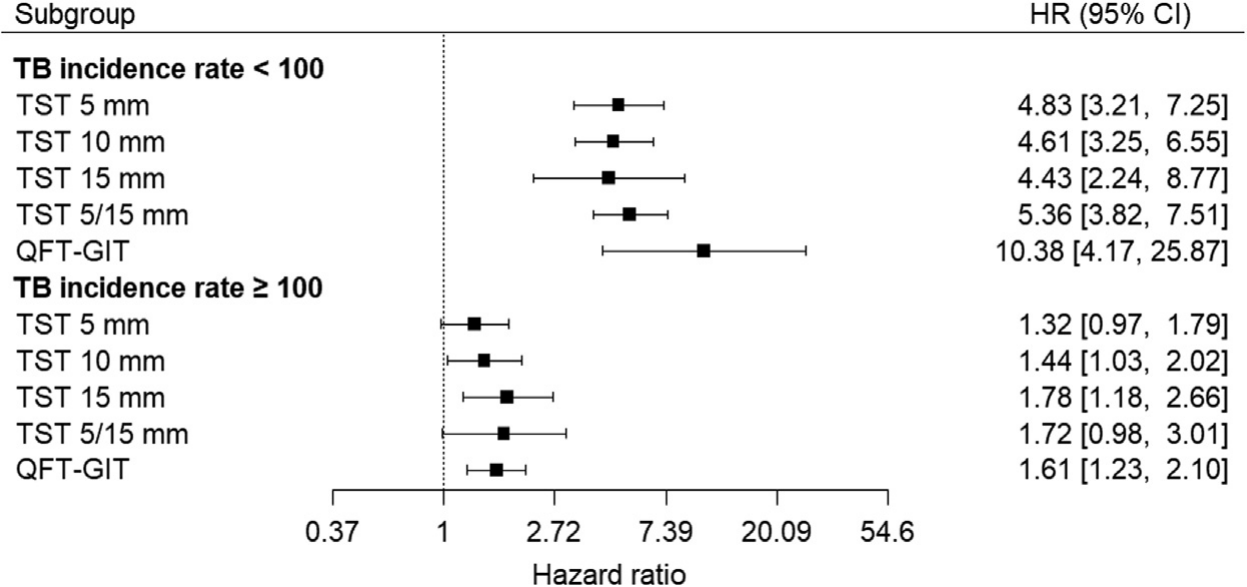
**Predictive performance of TST VS. QFT-GIT for all TB, by TB incidence in study countries.** TB: tuberculosis; HR: hazard ratio; CI: confidence interval; QFT-GIT: QuantiFERON Gold in Tube; TST: tuberculin skin test. HR indicates the risk of TB is participants with positive test results relative to those with negative results. In the original dataset before multiple imputation, studies in countries with TB incidence rate <100/100,000 and ≥100/100,000 population included 19,242 and 10,050 participants who did not receive TB preventive treatment, respectively. The analysis was performed using multiply imputed datasets in which missing data on the receipt of TB preventive treatment (n = 859) were imputed. When one study (Diel 2011) where HR could not be estimated directly was excluded from studies in TB incidence rate <100 per 100,000 populations, HR was 4.83 (95% CI 3.16–7.38) for TST 5 mm, 5.05 (95% CI 3.4–7.41) for TST10 mm, 5.86 (95% CI 4.17–8.22) for TST15 mm, 5.13 (95% CI 3.58–7.35) for TST5/15 mm, and 7.18 (95% CI 4.48–11.51) for QFT-GIT.

When we stratified data into two groups based on the TB incidence rate of the study country, the pooled predictive performance was higher among individuals in studies conducted in countries with TB incidence rates <100 per 100,000 population (Brazil, China and European countries). For example, the HR for TST^15 mm^ was 4.43 (95% CI 2.24–8.77) in countries with TB incidence rate <100 per 100,000 population compared to 1.78 (95% CI 1.18–2.66) in countries with TB incidence rate ≥100 per 100,000 population (India, South Africa and Zambia), respectively (Fig. 3). In countries with TB incidence rate <100 per 100,000 population, the point estimate for QFT-GIT was substantially higher (HR 10.38; 95% CI 4.17–25.87) with a wide confidence interval. The differences in the performance by setting were statistically significant (Table S9).

The exclusion of HIV-positive participants replicated the higher predictive performance in countries with low TB incidence than those with high TB incidence observed in the primary analysis (Fig. S7).

Figs. S8, S9, and Table S10 present the sensitivities and specificities for predicting the development of active TB over two years. None of the performances reached WHO minimum targets (>75% sensitivity and specificity). In low TB incidence countries, the performance was closer to the targets than in high TB incidence countries. In high TB incidence countries, the specificity for TST^5 mm^ was lower than in low TB incidence countries (44% VS. 51%), and so was the sensitivity (60% VS. 83%).

Based on the sensitivity and specificity estimates stratified by TB incidence, we estimated PPVs and NPVs in a hypothetical population of 10,000 individuals (Table S11). The median cumulative TB incidences were 0.95% and 5.2% for countries with TB incidence rate <100 and ≥100 per 100,000 population, respectively; thus, pre-test probability of TB incidence was assumed to be 1% and 5% over two years for each setting. In countries with TB incidence rate <100 per 100,000, PPV were numerically similar between TST^15 mm^ (2.75%) and QFT-GIT (2.46%) and so were NPV (99.42% VS. 99.52%), even though the overall performance (i.e. PPV/[100−NPV]) was slightly higher for QFT-GIT. In countries with TB incidence rate ≥100 per 100,000, the PPV for TST^15 mm^ was higher than for QFT-GIT (7.54% VS. 6.04%). NPV was lower for TST^15 mm^ than for QFT-GIT (95.66% VS. 96.26%).

All three studies using T-SPOT.TB were from European countries with TB incidence rate <100 per 100,000 populations; thus, sub-group analysis was not possible.

The predictive performance for TST tended to be lower in participants of older age (Table S9, Fig. S10). Hazard ratios were 2–3% lower for each 10-year increase in age. The same trend was not observed for QFT-GIT. This was in part driven by a study among household contacts in India (n = 1510),^24^ in which the HR for TB in children with positive QFT-GIT results was lower (HR 1.25, 95% CI 0.59–2.66) than that in adults (HR 2.46, 95% CI 1.18–5.15). In the same study, more children who did not develop TB disease had positive results for QFT-GIT (62.4%) than for TST^10 mm^ (45.6%), suggesting a lower specificity of QFT-GIT in this population.

We did not find a difference in predictive performance by BMI (Table S9). The estimates for the difference in predictive performance were imprecise with very wide confidence intervals for contact history (Table S8 and Fig. S11), or HIV status (Table S9). Six studies included both HIV-positive and negative individuals,^13^^,^^15^^,^^16^^,^^18^^,^^25^^,^^26^ but in four of them,^13^^,^^15^^,^^18^^,^^26^ HIV-positive individuals accounted for only <2%. There were no TB incident cases among HIV-positive individuals in one of the two remaining studies.^25^ Full results of the one-stage meta-analysis estimating interaction effects are presented in Table S9 and Table S12.

Aggregated data were most commonly available for TST^10 mm^ and QFT-GIT 10 studies, Fig. S12.^34^, ^35^, ^36^^,^^42^^,^^44^, ^45^, ^46^^,^^48^, ^49^, ^50^ When they were pooled with seven studies from low TB incidence countries that provided IPD, the pooled odds ratios were 4.20 (95% CI 2.71–6.49) for TST^10 mm^ and 12.00 (95% CI 4.46–32.30) for QFT-GIT (Fig. S13).

## Discussion

Our IPD meta-analysis found that QFT-GIT appeared to have higher predictive performance than the TST in the overall population. When stratified by national TB incidence, the difference was pronounced in low TB incidence countries, yet with wide confidence intervals. This is consistent with observations in previous head-to-head meta-analyses which showed higher point estimates for QFT-GIT but with overlapping wide CIs.^5^^,^^20^ The higher estimates observed in this IPD analysis were driven by a single study in a low TB incidence setting. In low TB incidence settings, test differences between PPV and NPV were minimal. This can be explained by two factors. First, studies reporting high predictive performance (e.g. Diel et al.) had less impact on the pooled estimates when sensitivity and specificity estimates are pooled because they are bounded between 0 and 100%. Second, pre-test probability for developing TB is low in low TB incidence settings. The minimal difference in PPV and NPV suggests that the higher performance does not appear to translate into a significant difference in clinical impact. On the other hand, the performance of TST and QFT-GIT was similar in high TB incidence countries. These findings support the current WHO guidelines that recommend using TST and IGRA interchangeably in instances where TB infection testing is recommended. Our IPD also found that the predictive performance of both tests was higher in low TB incidence countries—and approaching WHO targets of ≥75% for sensitivity (77% for TST^5/15 mm^ and 73% for QFT-GIT), but not specificity (62% for TST^5/15 mm^ and 65% for QFT-GIT)—and was lower in countries with high TB incidence.

The most recent review by Zhou et al. reported that the predictive performance of IGRA was almost twice as high as TST (pooled risk ratio of 9.35 VS. 4.25).^5^ All but one study included in the review were conducted in countries with TB incidence rate 100 < 100,000 populations. The higher predictive performance for IGRA in Zhou et al. was consistent with our estimates in low TB incidence countries despite the limitations in the previous review, such as the inclusion of non-head-to-head studies in the primary analysis, heterogeneity in TST cut-off values, and the use of effect measures not accounting for the follow-up duration.^6^ However, the IPD approach shows that the effect estimates remain imprecise, and the added prognostic advantage of IGRA over the TST may still be small.

In contrast to previous aggregated-data meta-analyses, we were able to assess the difference in the predictive performance by background TB incidence, using the standardised TST cut-off values and the definition of TB, which showed a lower predictive performance in high TB incidence countries. Differences in TB epidemiology could explain this finding. First, in countries with high TB incidence, pre-test probability of developing TB is higher than in countries with low TB incidence, which results in lower NPV even if the sensitivity and specificity of the tests do not differ. Moreover, more people with negative test results at baseline may get infection and develop TB, which lowers the sensitivity. In that case, the actual performance to predict progression to active TB from infection might be higher than estimated. Second, in high TB incidence countries, people would have more chances to be infected early in their life before they are identified as contacts. As a result, a large subset of positive results at baseline may reflect the evidence of remote exposure rather than recent infection that is likely to progress. This lowers the specificity and predictive performance of tests for TB infection. The poorer performance in high TB incidence countries calls for an urgent need for a better test or multivariable clinical algorithm for predicting the development of TB in settings which have the greatest burden of disease.^54^ While mRNA signatures have shown promising results in early studies,^55^^,^^56^ they have suboptimal performance for long-term prediction over two years and are not yet available commercially. In the meantime, the role of TB infection tests needs to be carefully considered in light of the trade-off between false positives and negatives and their clinical and public health impact. For instance, preventive treatment could be given without tests in populations who are at very high risk for TB, especially those at high risk for severe diseases such as young children and people living with HIV, as already recommended by WHO.^1^ On the contrary, tests can reduce the number of people who are started on preventive treatment and reduce opportunity costs. This would be particularly important in populations who are at risk for adverse events. Furthermore, consideration of quantitative results as well as sociodemographic and clinical factors might enable a more accurate estimate of the risk of TB and individualised management, currently being done in high-income, low-incidence contexts.^10^^,^^54^

Our review intended to conduct *a priori* defined subgroup analyses. We found that the predictive performance for TST tended to be higher in younger populations. This might reflect that in younger populations, positive results can be better explained by recent infection. Interestingly, the same trend was not observed for QFT-GIT. The difference in the trend might be explained by the declining sensitivity of TST in older adults reported in previous studies.^57^ On the other hand, the lack of the trend for QFT-GIT was affected by a single study in which the test had a lower predictive performance in children than adults.^24^ However, given the small number of events and multiple analyses, this observation may be a chance finding. Furthermore, since we could not fit multivariable models due to insufficient sample size and event rate, and we did not derive a specific causal model, the presence of confounding might also explain these findings. Other subgroup analyses had low statistical power, and some risk groups—such as individuals with co-morbidities (e.g. diabetes, autoimmune diseases, or HIV), of advanced age, or receiving immunosuppressants or chemotherapy—were not considered due to limited data, currently limiting our understanding of the impact of those covariates. Likewise, a low sample size of individuals <18 years old precluded disaggregation of individuals into children and adolescents in the sub-groups analysis. About a third of the study proportion were individuals who do not belong to specific risk groups (adolescents in South Africa and general adults in China), who are at lower risk for disease progression. The performance is likely to differ if restricted to those at high risk. In addition, we did not consider the impact of socioeconomic status (e.g. living environment and education level) due to limited availability and difficulty in standardizing those variables.

None of the included studies blinded results of TB infection tests for diagnosing incident TB. Thus, the knowledge of TB infection test results might have affected the ascertainment of incident TB and then overestimated the predictive performance (i.e. incorporation bias). This impact might be stronger for IGRA if clinicians believed that IGRA was more accurate or there was differential work-up for positive results than negative ones.

The median (IQR) follow-up duration in our study was 3.6 (2.0–5.6) years, which may have not been long enough for assessing incident TB. Nevertheless, WHO's targets for tests for predicting TB development are set for two-year time horizon, given that the risk is highest shortly after infection.^58^ We were able to compare the performance of TST and IGRA with these targets.

We could not obtain data from 27 of 40 studies that were considered eligible. Nonetheless, we were able to include data from 32,034 participants, more than the 20,333 from the remaining studies, corresponding to >60% of the total participants. The sample size is the largest for a head-to-head analysis of TST VS. IGRA, representing both high and low TB incident settings, compared to 4875, mostly from low TB incidence countries, included in the most recent review.^5^ Furthermore, our sensitivity analysis that included aggregated data from studies without IPD consistently showed a higher predictive performance for IGRA than TST in low TB incidence countries.

Lastly, we are aware of other IGRA kits than QFT-GIT and TSPOT.TB.^59^ We did not include them due to lack of data on their predictive performance. Some of those tests have been suggested to give results that are highly concordant with the existing tests.^59^ For those tests that have a high level of agreement with QFT-GIT and/or TSPOT.TB, the results of our analysis are likely to be applicable.

Our IPD meta-analysis found that IGRA appeared to have higher performance than TST for predicting the development of TB disease in countries with low TB incidence. However, the difference was driven by a single study and does not appear to make a large difference in clinical impact. Both tests had performance close to minimum WHO targets in low TB incidence countries, whereas performance was poorer in high TB incidence countries. This reinforces the urgent need for a better test to predict the development of TB disease. While we wait for a new test, test choice needs to be made considering operational and clinical impact, and alternative strategies such as the use of quantitative results combined with demographic and clinical factors should be explored.

## Contributors

YH, RKG, MQ, IA, and MXR designed the study and protocol and interpreted the results. YH, RKG, and AI did the systematic review. CAV, NA, RD, JD, SF, AG, HH, ECJ, AK. CL, FvL, QL, WL, PL, ILR, LMa, SKM, LMu, ESP, MP, TS, MS, KS, SKS, RS, GS, KT, RV, and IA contributed data to the meta-analysis. YH analysed the data with assistance from RKG and QM. YH wrote the first draft of the manuscript, which was revised based on comments from co-authors. YH and RKG accessed and verified the data. All authors approved the final version of the manuscript.

## Data sharing statement

The IPD database will be stored within the UCL Data Repository and can be shared subject to the approval of the corresponding authors of the original studies.

## Declaration of interests

YH and MXR report donation of QIAreach, an IGRA, by QIAGEN for a LTBI infection survey. YH, MXR, and IA report donations of Cy-TB, a TB-specific skin test for detection of LTBI, by the Serum Institute of India for a study on the feasibility and patient-important outcomes. They had no role in the submitted work. RD declared the receipt of payment for lectures from Qiagen and Oxford Immunotec. JD declared honoraria for lectures from Oxford Immunotec. AG declared receipt of research grants from the US National Institute of Health (NIH) and membership in NIH Council and IndoUS Science Technology Forum. CL provided consultation service to INSMED and received speakers honoraria from INSMED, GILEAD, JANSSEN and is a member of the Data Safety Board of trials from Medicines sans Frontiers, all outside of the submitted work. ILR has a patent (PCT/EP2019/064885), *in vitro* method for the diagnosis or detection of non-tuberculous mycobacteria. MS reports receipt of test kits free of charge from Qiagen and Oxford Immunotec. MS also reports receipt of research grants from Biotest and Astellas, consulting fees and honoraria from Biotest, Moderna, Qiagen, and Takeda, support for travel from Biotest, and participation on advisory board for Biotest and Moderna, all outside of this work. GS reports receipt of consulting fees from Pfizer, Diasorin, and INSMED. All other authors declare no competing interests.

## Appendix A Supplementary data

Supplementary_9Nov2022-R3
