# Supplementary material for: Predictive performance of interferon-gamma release assays and the tuberculin skin test for incident tuberculosis: an individual participant data meta-analysis
Source: eClinicalMedicine. 2023 Jan 5;56:101815. doi: 10.1016/j.eclinm.2022.101815 (PMC9829704; doi:10.1016/j.eclinm.2022.101815)
Supplement: Supplementary_9Nov2022-R3 [file mmc1.docx]

Contents

[Appendix 1. Supplementary methods 2](#_Toc121943123)

[Appendix 2. Search strategy for Medline search 6](#_Toc121943124)

[Appendix 3. List of variables requested 7](#_Toc121943125)

[Appendix 4. Quality assessment criteria 8](#_Toc121943126)

[Appendix 5. Look-up table for transformation from raw TB infection test results to normalised percentile scale 9](#_Toc121943127)

[Appendix 6 Supplementary tables and figures 10](#_Toc121943128)

[Table A2. Characteristics of studies that did not provide individual participant data 11](#_Toc121943129)

[Table A3. Study characteristics 12](#_Toc121943130)

[Table A4. Baseline characteristics by study 14](#_Toc121943131)

[Table A5. Quality assessment of included studies 16](#_Toc121943132)

[Figure A1. Cumulative incidence of TB stratified by test results 17](#_Toc121943133)

[Figure A2. Forest plots of the predictive performance of TST vs QFT-GIT for all TB 18](#_Toc121943134)

[Figure A3. Predictive performance of TST vs QFT-GIT for all TB using two-stage Poission regression meta-analysis 19](#_Toc121943135)

[Table A6. Comparison of the predictive performance of TST vs IGRA for all TB 20](#_Toc121943136)

[Figure A4. Predictive performance of TST vs TSPOT.TB for all TB 21](#_Toc121943137)

[Figure A5. Predictive performance of TST vs TSPOT.TB for all TB using two-stage Poission regression meta-analysis 22](#_Toc121943138)

[Table A7. Pooled estimates of the predictive performance of TST vs QFT-GIT-sensitivity analysis 23](#_Toc121943139)

[Table A8. Pooled estimates of the predictive performance of TST vs TSPOT.TB-sensitivity analysis 24](#_Toc121943140)

[Figure A6. Funnel plots and p values for Egger’s test 25](#_Toc121943141)

[Table A9. The differences in the predictive performance of TST and QFT-GIT for all TB- the ratio of hazard ratios 26](#_Toc121943142)

[Figure A7. Predictive performance of TST vs QFT-GIT for all TB by TB incidence in study countries, excluding HIV-positive participants 27](#_Toc121943143)

[Figure A8. Sensitivity and specificity for predicting the development of active TB over two years 28](#_Toc121943144)

[Figure A9. Sensitivity and specificity for predicting the development of active TB over two years- studies reporting TSPOT.TB 29](#_Toc121943145)

[Table A10. Sensitivity and specificity for predicting the development of active TB over two years 30](#_Toc121943146)

[Table A11. Positive and negative predictive value in a hypothetical population (n = 10,000) 31](#_Toc121943147)

[Figure A10. Predictive performance of TST vs QFT-GIT for all TB, Adults vs Children and adolescents 32](#_Toc121943148)

[Figure A11. Predictive performance of TST vs QFT-GIT for all TB by contact history 33](#_Toc121943149)

[Table A12. The differences in the predictive performance in studies with TSPOT.TB data- the ratio of hazard ratios 34](#_Toc121943150)

[Figure A12. Studies that did not provide IPD 35](#_Toc121943151)

[Figure A13. Meta-analysis of the predictive performance of TST vs QFT-GIT for all TB stratified by the availability of IPD 36](#_Toc121943152)

# Appendix 1. Supplementary methods

**Handling of missing data**

We excluded participants with missing follow-up durations. Most (95%) of them lacked outcome data and contributed little information. We assumed Missing at Random (MAR) given that all the available information was the most likely missingness mechanism for the remaining observations; hence, we conducted multiple imputations using multilevel fully conditional specification accounting for clustering by study to impute incident TB outcomes, index test results, and covariates. The models included the outcome (tuberculosis [TB] disease), test results (tuberculin skin tests [TST], QuantiFERON Gold in-Tube [QFT-GIT], and TSPOT), variables used for sub-group analysis (age, TB disease incidence in study countries, contact history, HIV status, and body mass index [BMI]) and auxiliary variables, including previous bacillus Calmette–Guérin vaccine (BCG) vaccination, previous TB, smoking, preventive TB treatment. We created interaction terms between test results and sub-grouping variables before imputations and added them as "just another variable", since no method for imputing compatibly with a Cox mixed effect model was available. We first conducted multiple imputations using binary test results, and the outputs were used to estimate the predictive performance of binary test results and their interactions with the above variables while ensuring congeniality. BMI was transformed first and then imputed (i.e., just another variable approach), but it was not used as a predictor for other variables to avoid correlation with height and weight. The Nelson-Aalen estimator was also included as a predictor.^1^

We generated 20 multiply imputed data sets with 20 iterations between successive imputations. Model convergence was assessed visually. All primary analyses were performed across multiply imputed datasets; substantive models were fitted on each imputed dataset, and their outputs were combined using Rubin's rules.^2^

**Predictive performance by population and setting**

We intended to present the predictive performance of tests using binary results by (a) age (adults [≥ 18 years old] VS. children and adolescents [< 18 years old]); (b) exposure group (contacts VS. non-contacts); (c) world region (TB incidence rate < 100 VS. ≥ 100 per 100,000 population); (d) HIV status, and, (5) BMI (categorized to overweight (BMI ≥25 kg/m^2^), normal weight (BMI ≥ 18.5 kg/m^2^ and < 25 kg/m^2^) and underweight (BMI < 18.5 kg/m^2^). Some studies included only one sub-group (i.e., contacts or HIV only), or some sub-groups accounted for only small proportions with few or no events, which resulted in non-convergence of the modelling. Where possible, we presented forest plots by sub-group restricting to studies that included all groups to allow within-study comparison. In addition, we fitted the models by including interaction terms for each sub-grouping variable separately. The models separated within-study and between-study interactions by centring the covariate about the mean and including the mean as an additional adjustment term.^15^ We also conducted regression models including age and BMI as continuous variables, respectively.

We estimated the sensitivity and specificity of tests to predict TB disease incidence over two years by setting and compared them against the minimum performance targets of > 75% sensitivity and specificity set by WHO.^3^ We first pooled sensitivities and specificities using a bivariate model for each imputed data set. We then combined their outputs using Rubin's rules. To explore the clinical impact, we presented positive and negative predictive values (PPV and NPV, respectively) in hypothetical populations with different pre-test probabilities of developing TB disease.

***Sensitivity analyses***

We repeated the analyses: 1) using a two-stage meta-analysis fitting Poisson regression models to estimate incident rate ratios for TB; (2) using shorter (<14 days) and longer (<180 days) temporal definitions of prevalent TB disease; and (3) including only incident TB disease (< 42 days) events with microbiological confirmation. Furthermore, we repeated sub-group analysis by TB incidence by excluding HIV-positive individuals to assess the impact of confounding by HIV status.

For the two-stage meta-analysis, we first estimated incident rate ratios in each study based on multiply imputed data sets. The results were pooled using the inverse variance method with the restricted maximum-likelihood estimator and Hartung-Knapp adjustment.^4^ The two-stage analysis showed results that are numerically different from the primary analysis. Thus, we conducted a sensitivity analysis by excluding a study with zero events in one of the groups with positive or negative test results by tests for TB infections in the one-stage meta-analysis to explore the source of the difference.

In order to assess the impact of studies that did not provide IPD, we combined their aggregated data with those studies with individual participant data (IPD) and conducted meta-analyses. We extracted the numbers of people with positive and negative test results and TB cases who were not given TB preventive treatment. From studies with IPD, we used data before multiple imputations to calculate the same numbers as above in participants not given TB preventive treatment. In studies without IPD, TST 10 mm and QFT-GIT were most commonly used, and data on other cut-off values and TSPOT.TB were limited. Therefore, we conducted meta-analyses for TST 10 mm and QFT-GIT. We estimated pooled odds ratio using mixed effects logistic regression models, combining all studies as well as stratified by the availability of IPD.

**References**

1. White IR, Royston P. Imputing missing covariate values for the Cox model. *Stat Med* 2009; **28**(15): 1982-98.

2. Rubin, D. B. Multiple Imputation for Nonresponse In Surveys (Wiley-Interscience, 2004).

3. World Health Organization. Consensus Meeting Report: Development of a Target Product Profile (TPP) and a framework for evaluation for a test for predicting progression from tuberculosis infection to active disease. Geneva, Switzerland: WHO, 2017.

4. IntHout J, Ioannidis JPA, Borm GF. The Hartung-Knapp-Sidik-Jonkman method for random effects meta-analysis is straightforward and considerably outperforms the standard DerSimonian-Laird method. *BMC Medical Research Methodology* 2014; **14**(1): 25.

5. Abubakar I, Drobniewski F, Southern J, et al. Prognostic value of interferon-γ release assays and tuberculin skin test in predicting the development of active tuberculosis (UK PREDICT TB): a prospective cohort study. *The Lancet Infectious Diseases* 2018; **18**(10): 1077-87.

# Appendix 2. Search strategy for Medline search

| 1. exp TUBERCULOSIS/ or tuberculosis.mp. or exp MYCOBACTERIUM TUBERCULOSIS/ or tb.mp. |
| --- |
| 2. exp Interferon-gamma Release Tests/ |
| 3. interferon gamma release.mp. |
| 4. igra.mp. |
| 5. t-spot*.mp. |
| 6. tspot*.mp. |
| 7. quantiferon*.mp. |
| 8. qft*.mp. |
| 9. 2 or 3 or 4 or 5 or 6 or 7 or 8 |
| 10. exp TUBERCULIN TEST/ |
| 11. tuberculin skin test.mp. |
| 12. tst.mp. |
| 13. purified protein derivative.mp. |
| 14. ppd.mp. |
| 15. mantoux.mp. |
| 16. 10 or 11 or 12 or 13 or 14 or 15 |
| 17. exp latent tuberculosis/ |
| 18. latent.mp. |
| 19. latent.mp. |
| 20. 9 or 16 or 19 |
| 21. exp "Predictive Value of Tests"/ |
| 22. predict*.mp. |
| 23. prognos*.mp. |
| 24. progress*.mp. |
| 25. ((tb adj2 developed) or (tuberculosis adj2 developed)).mp. |
| 26. ppv.mp. |
| 27. npv.mp. |
| 28. (incidence rate ratio or irr).mp. |
| 29. 21 or 22 or 23 or 24 or 25 or 26 or 27 or 28 |
| 30. 1 and 20 and 29 |
| 31. limit 30 to yr="2002 -2020" |
| 32. limit 31 to "humans only (removes records about animals)" |
| 33. remove duplicates from 32 |

# Appendix 3. List of variables requested

| **Category** | **Variables** |
| --- | --- |
| Demographics | Age, gender, ethnicity, occupation |
| Exposure to TB | Country of birth, year of migration, recent travel, presence/absence of recent contact with index case, contact proximity (e.g. household / non-household), date of index case diagnosis, contact duration, index case site of disease, index case sputum smear status, index case drug susceptibility. |
| Clinical risk factors | BMI, History of diabetes, transplantation, renal failure, cancer, anti-TNF therapy, corticosteroid exposure, silicosis, HIV, smoking, problem drug use, alcohol excess, history of homelessness, imprisonment, BCG vaccination history, previous TB history |
| LTBI screening | IGRA type, date and result (binary and quantitative), TST date and result, LTBI treatment acceptance and completion |
| Outcome | Date of TB diagnosis, date of death, date of follow-up censor |

TB: tuberculosis; LTBI: latent tuberculosis infection; BMI: body mass index; HIV: human immunodeficiency virus; BCG: bacillus Calmette–Guérin vaccine; TNF: tumor necrosis factor; IGRA: interferon-gamma release assay; TST: tuberculin skin test

# Appendix 4. Quality assessment criteria

Ø indicates criteria to score a maximum of one point for each domain.

| **Selection** |
| --- |
| 1) Representativeness of the exposed cohort |
| a) truly representative of the target population in the community Ø |
| b) somewhat representative of the target population in the community Ø |
| ^5^c) selected group of users eg nurses, volunteers |
| d) no description of the derivation of the cohort |
|  |
| 2) Selection of the non exposed cohort |
| a) drawn from the same community as the exposed cohort Ø |
| b) drawn from a different source |
| c) no description of the derivation of the non exposed cohort |
|  |
| 3) Ascertainment of LTBI test result |
| a) secure record Ø |
| b) structured interview Ø |
| c) written self report |
| d) no description |
|  |
| 4) Assessment for prevalent TB at start of study |
| a) yes Ø |
| b) no |
|  |
| **Outcome** |
| 1) Assessment of outcome |
| a) independent assessment blinded to LTBI test result Ø Ø |
| b) data available on microbiological confirmation Ø |
| c) record linkage Ø |
| d) self report |
| e) no description |
|  |
| 2) Was follow-up long enough for outcomes to occur |
| a) yes (median >1 year) Ø |
| b) no |
|  |
| 3) Adequacy of follow up of cohorts |
| a) complete follow up - all subjects accounted for Ø |
| b) subjects lost to follow up unlikely to introduce biasØ:  - small number lost (<5%); - or characteristics of those lost similar to those followed-up |
| c) follow up rate < 95% and no description of those lost |
| d) no statement |
|  |
| Total (maximum 8) |

TB: tuberculosis; LTBI: latent tuberculosis infection

# Appendix 5. Look-up table for transformation from raw TB infection test results to normalised percentile scale

| Percentile | TST (mm) | QFT-GIT (IU/ml) | TSPOT.TB (spots) |
| --- | --- | --- | --- |
| 1 | 0 to 1 | 0 to 0 | 0 to 0 |
| 30 |  | 0.006 to 0.009 |  |
| 31 |  |  |  |
| 32 |  |  |  |
| 33 |  |  |  |
| 34 |  |  |  |
| 35 |  |  |  |
| 36 |  | 0.01 to 0.019 |  |
| 37 |  |  |  |
| 38 |  |  |  |
| 39 |  |  |  |
| 40 |  | 0.02 to 0.029 |  |
| 41 |  |  |  |
| 42 |  | 0.03 to 0.039 |  |
| 43 |  |  |  |
| 44 | 2 to 2 | 0.04 to 0.049 |  |
| 45 | 3 to 3 |  |  |
| 46 |  | 0.05 to 0.059 |  |
| 47 | 4 to 4 | 0.06 to 0.069 |  |
| 48 |  | 0.07 to 0.079 |  |
| 49 |  | 0.08 to 0.089 |  |
| 50 | 5 to 5 | 0.09 to 0.099 | 1 to 1 |
| 51 |  | 0.1 to 0.109 |  |
| 52 |  | 0.11 to 0.119 |  |
| 53 | 6 to 6 | 0.12 to 0.139 |  |
| 54 |  | 0.14 to 0.159 |  |
| 55 | 7 to 7 | 0.16 to 0.179 |  |
| 56 |  | 0.18 to 0.199 |  |
| 57 |  | 0.2 to 0.229 |  |
| 58 | 8 to 8 | 0.23to 0.259 |  |
| 59 |  | 0.26 to 0.289 |  |
| 60 |  | 0.29 to 0.319 |  |
| 61 | 9 to 9 | 0.32 to 0.349 |  |
| 62 |  | 0.35 to 0.409 |  |
| 63 |  | 0.41 to 0.459 |  |
| 64 |  | 0.46 to 0.539 |  |
| 65 |  | 0.54 to 0.619 | 2 to 2 |
| 66 | 10 to 10 | 0.62 to 0.699 |  |
| 67 |  | 0.7 to 0.799 |  |
| 68 |  | 0.8 to 0.899 |  |
| 69 | 11 to 11 | 0.9 to 1.019 |  |
| 70 |  | 1.02 to 1.129 |  |
| 71 |  | 1.13 to 1.289 | 3 to 3 |
| 72 |  | 1.29 to 1.429 |  |
| 73 | 12 to 12 | 1.43 to 1.549 |  |
| 74 |  | 1.55 to 1.679 |  |
| 75 |  | 1.68 to 1.829 | 4 to 4 |
| 76 | 13 to 13 | 1.83 to 1.975 |  |
| 77 |  | 1.976 to 2.179 | 5 to 5 |
| 78 |  | 2.18 to 2.449 |  |
| 79 | 14 to 14 | 2.45 to 2.709 | 6 to 6 |
| 80 |  | 2.71 to 2.989 | 7 to 7 |
| 81 |  | 2.99 to 3.339 | 8 to8 |
| 82 |  | 3.34 to 3.769 | 9 to 9 |
| 83 | 15 to 15 | 3.77 to 4.269 | 10 to 11 |
| 84 |  | 4.27 to 4.809 | 12 to 13 |
| 85 |  | 4.81 to 5.439 | 14 to 15 |
| 86 | 16 to 16 | 5.44 to 6.209 | 16 to 17 |
| 87 |  | 6.21 to 7.089 | 18 to 20 |
| 88 | 17 to 17 | 7.09 to 7.969 | 21 to 23 |
| 89 |  | 7.97 to 8.909 | 24 to 27 |
| 90 |  | 8.91 to 9.829 | 28 to 32 |
| 91 | 18 to 18 | 9.83 to 9.99 | 33 to 38 |
| 92 | 19 to 19 |  | 39 to 45 |
| 93 |  |  | 46 to 53 |
| 94 |  |  | 54 to 64 |
| 95 | 20 to 20 |  | 65 to 77 |
| 96 | 21 to 21 |  | 78 to 88 |
| 97 | 22 to 24 |  | 99 to 127 |
| 98 | 25 to 27 |  | 128 to 174 |
| 99 | ≥28 | ≥10 | ≥ 175 |

# Appendix 6 Supplementary tables and figures

**Table A1. Eligibility criteria**

| **Inclusion criteria** |
| --- |
| 1. Studies that tested participants with TST and IGRA and followed them up for development of incident TB |
| 1. Prospective study design |
| 1. Minimum median duration of follow-up one year. |
| 1. Conducted in any country. |
| 1. TST and at least one commercial IGRA performed among study participants. |
| 1. Minimum individual level exposure variables recorded should include age, gender, indication for LTBI screening, result of LTBI screening test (positive or negative), and whether preventative therapy was provided. |
| 1. Minimum individual level outcome variables recorded should include presence or absence of active TB during follow-up, date of TB diagnosis, date of follow-up censor. |
| 1. Study participants should be recruited after 1^st^ January 2001. |
| **Exclusion criteria** |
| 1. Data are not available for calculation of relative risks for TB |
| 1. TPT was given differentially by indication to test positives and all participants with positive LTBI tests received TPT |
| 1. Studies using a '2-step' strategy where only participants with a positive first test are eligible for testing with the second test. |
| 1. Studies limited to single contact investigations (a single outbreak investigation). |
| 1. Studies without associated full-text publications. |

TB: tuberculosis; LTBI: latent tuberculosis infection; TPT: tuberculosis preventive treatment ; IGRA: interferon-gamma release assay; TST: tuberculin skin test

## Table A2. Characteristics of studies that did not provide individual participant data

|  | **Study** | **Country** | **Study population** | **Age** |
| --- | --- | --- | --- | --- |
|  | Ahmed, 2010 | United States | Children eligible for treatment of TB infection | Median: 8.6 years old |
|  | Bergot, 2012 | France | Close contacts | Median: 42 years old |
|  | Bourgarit, 2015 | France | People living with HIV | Mean: 39 years old |
|  | Debulpaep, 2019 | Belgium | Close contacts | < 5 years old |
|  | Dorman, 2014 | United States | Health care workers | Median: 36.2 years old |
|  | Harstad, 2010 | Norway | Asylum seekers | ≥ 18 years old |
|  | Joshi, 2011 | India | Health care workers | > 0 years old |
|  | Jung, 2012 | South Korea | Anti-TNF recipient | Mean: 40 years old |
|  | Kim, 2013 | South Korea | Kidney transplant recipients | Mean: 45 years old |
|  | Kruczak, 2014 | Poland | Homeless persons, contacts, and residents in long-term care facilities | Median: 49 years old |
|  | Lee, 2009 | Taiwan | Patients with end-stage renal disease and healthy adults | Mean: 54 years old |
|  | Lee, 2014 | South Korea | Transplant recipients | Mean: 42 years old |
|  | Leung, 2010 | South Korea | Male with silicosis | Mean: 60 years old |
|  | Leung, 2015 | South Korea | Household contacts | Mean: 30 years old |
|  | Mathad, 2016 | India | HIV- positive women pre-and post-partum | ≥ 18 years old |
|  | McCarthy, 2015 | South Africa | Health care workers and medical students | Median: 22 years old for medical students and 33 years old for health care workers |
|  | Noorbakhsh,2011 | Iran | Household contacts | < 20 years old |
|  | Pullar, 2014 | Norway | People living with HIV | > 18 years old |
|  | Ringshausen, 2010 | Germany | Health care workers | Median: 38 years old |
|  | Seyhan, 2016 | Turkey | Patients on haemodialysis and healthy adults | Mean: 58 and 51 years old, respectively |
|  | Song, 2014 | South Korea | Close contacts | Mean: 15 years old |
|  | Torres Costa, 2011 | Portugal | Health care workers | Not reported |
|  | Tsou, 2015 | Taiwan | Nursing home residents | <25 years: 10.4%  25-29 years: 28.5%  30-39 years: 27.4%  40-49 years: 18.5%  50 years ≥: 15.2% |
|  | Verhagen, 2014 | Venezuela | Household contacts | Mean: 7.7 years old |
|  | Yang, 2013 | Taiwan | People living with HIV | Median: 35 years old |
|  | Zhang, 2019 | China | Household contacts | ≥ 14 years old |
|  | Zhang, 2013 | China | Health care workers | Median: 26 years old |

QFT-GIT: QuantiFERON Gold in Tube; TST: tuberculin skin test; TNF’ tumour-necrosis factor

## Table A3. Study characteristics

| Study | Country | Study population | IGRA used | Methods for exclusion of active TB at baseline | Methods to ascertain incident TB | Definition of active TB |
| --- | --- | --- | --- | --- | --- | --- |
| Abubakar 2018 | UK | Contacts and migrants from high TB incidence countries | QFT-GIT, T-SPOT.TB | Symptom and chest x-ray | Record linkage | Bacteriologically confirmed and clinically diagnosed (radiological or histological evidence and receipt of TB treatment) |
| Acuna-Villaorduna 2018 | Brazil | Household contacts | QFT-GIT | In accordance with national guidelines (symptom screening and chest x-ray) | Record linkage | Bacteriologically confirmed |
| Altet 2015 | Spain | Close contacts | QFT-GIT, T-SPOT.TB | Chest x-ray in all | Clinical screening at every visit and reviewing the TB Programme database | Bacteriologically confirmed and clinically diagnosed (no details) |
| Diel 2011 | Germany | Close contacts | QFT-GIT | Not reported | Record linkage | Bacteriologically confirmed and clinically diagnosed (symptoms and x-rays) |
| Huerga 2019 | Armenia | Close contacts | QFT-GIT | Symptom screening and chest x-ray in all. | Symptom screening and chest x-ray at follow-up visits. | Bacteriologically confirmed and clinically diagnosed (symptoms and x-rays) |
| Lu 2021 | China | General population (≥ 5 years old) | QFT-GIT | Symptom screening and chest x-ray in all participants ≥15 years and in those < 15 years with TB symptoms or history of close contact | Symptom screening at every 3 months and record linkage. | Bacteriologically confirmed and clinically diagnosed (symptoms, x-rays, and response to treatment) |
| Mahomed 2011 | South Africa | HIV-negative adolescents | QFT-GIT | Participants with symptoms, positive TB infection tests or contacts referred for two sputum smears | Symptom screening at every visit. Surveillance of clinic and hospital TB records. | Bacteriologically confirmed only |
| Muñoz 2015 | Spain | Liver or stem cell transplant recipients | QFT-GIT | Symptoms and chest x-ray in all. | Review of medical records and contacting treating physicians | Bacteriologically confirmed only |
| Paradkar 2020 | India | Household contacts | QFT-GIT | Chest x-ray and sputum tests smear, Xpert and culture in all | Screening of signs and symptoms at every visit followed bacteriological tests | Both bacteriologically confirmed and clinically diagnosed (receipt of empirical treatment based on clinical and/or x-ray findings). |
| Rangaka 2014 | South Africa | People living with HIV in a randomized controlled trial | QFT-GIT | Symptom screening followed by confirmatory tests | Symptoms screening followed by bacteriological tests at each study visit. Supplemented by record linkage. | Both bacteriologically confirmed and clinically diagnosed (clinical findings with x-ray). |
| Sester 2014 | 11 European countries | Immunocompromised adults (people living with HIV, chronic renal failure, rheumatoid arthritis, solid-organ or stem-cell transplantation. | QFT-GIT, T-SPOT.TB | No information | “Actively sought and collected by personal patient contact by the treating physician.” | Both bacteriologically confirmed and clinically diagnosed (signs and symptoms and treatment response). |
| Shanaube 2014 | South Africa and Zambia | Household contacts (including HIV-positive and negative) | QFT-GIT | Not defined | Self-report confirmed in TB registers. | Bacteriologically confirmed and clinically diagnosed (based on treatment cards) |
| Sharma 2017 | India | Household contacts | QFT-GIT | Symptom screening followed by chest x-ray and by confirmatory tests | Symptom screening at every visits, followed by chest x-ray and bacteriological tests. | Both bacteriologically confirmed and clinically diagnosed (basis of imaging or presence of exudative effusion or other body fluids with elevated adenosine deaminase activity |

TB: tuberculosis; QFT-GIT: QuantiFERON Gold in Tube; TST: tuberculin skin test

## Table A4. Baseline characteristics by study

|  | **Abubakar 2018** | **Acuna-Villaorduna 2018** | **Altet 2015** | **Diel 2011** | **Huerga 2019** | **Lu 2021** | **Mahomed 2011** | **Munoz 2015** | **Paradkar 2020** | **Rangaka 2014** | **Sester 2014** | **Shanaube 2014** | **Sharma 2017** |
| --- | --- | --- | --- | --- | --- | --- | --- | --- | --- | --- | --- | --- | --- |
| N | 9826 | 890 | 1258 | 1414 | 148 | 5404 | 6363 | 76 | 1009 | 1324 | 1462 | 1350 | 1510 |
| Age (mean (SD)) | 36.97 (15.20) | 26.06 (19.67) | 24.43 (15.30) | 29.97 (11.81) | 6.19 (4.20) | 49.70 (17.65) | 15.25 (1.50) | 54.32 (10.16) | 27.34 (16.34) | 34.81 (8.32) | 49.59 (15.13) | 33.71 (15.64) | 24.33 (15.18) |
| Gender (%) |  |  |  |  |  |  |  |  |  |  |  |  |  |
| Female | 4898 ( 49.8) | 494 ( 55.5) | 623 ( 49.5) | 684 ( 48.4) | 79 ( 53.4) | 2873 ( 53.2) | 3458 ( 54.3) | 30 ( 39.5) | 561 ( 55.6) | 996 ( 75.2) | 548 ( 37.5) | 966 ( 71.6) | 726 ( 48.1) |
| Missing | 72 ( 0.7) | 0 ( 0.0) | 1 ( 0.1) | 0 ( 0.0) | 0 ( 0.0) | 0 ( 0.0) | 0 ( 0.0) | 0 ( 0.0) | 0 ( 0.0) | 0 ( 0.0) | 0 ( 0.0) | 0 ( 0.0) | 0 ( 0.0) |
| Reason for screening (%) |  |  |  |  |  |  |  |  |  |  |  |  |  |
| Contact | 4985 ( 50.7) | 890 (100.0) | 1258 (100.0) | 1414 (100.0) | 148 (100.0) | 0 ( 0.0) | 0 ( 0.0) | 0 ( 0.0) | 1009 (100.0) | 0 ( 0.0) | 0 ( 0.0) | 1350 (100.0) | 1510 (100.0) |
| Immunosuppression | 0 ( 0.0) | 0 ( 0.0) | 0 ( 0.0) | 0 ( 0.0) | 0 ( 0.0) | 0 ( 0.0) | 0 ( 0.0) | 76 (100.0) | 0 ( 0.0) | 1324 (100.0) | 1462 (100.0) | 0 ( 0.0) | 0 ( 0.0) |
| Migrant | 4739 ( 48.2) | 0 ( 0.0) | 0 ( 0.0) | 0 ( 0.0) | 0 ( 0.0) | 0 ( 0.0) | 0 ( 0.0) | 0 ( 0.0) | 0 ( 0.0) | 0 ( 0.0) | 0 ( 0.0) | 0 ( 0.0) | 0 ( 0.0) |
| Others | 0 ( 0.0) | 0 ( 0.0) | 0 ( 0.0) | 0 ( 0.0) | 0 ( 0.0) | 5404 (100.0) | 6363 (100.0) | 0 ( 0.0) | 0 ( 0.0) | 0 ( 0.0) | 0 ( 0.0) | 0 ( 0.0) | 0 ( 0.0) |
| Missing | 102 ( 1.0) | 0 ( 0.0) | 0 ( 0.0) | 0 ( 0.0) | 0 ( 0.0) | 0 ( 0.0) | 0 ( 0.0) | 0 ( 0.0) | 0 ( 0.0) | 0 ( 0.0) | 0 ( 0.0) | 0 ( 0.0) | 0 ( 0.0) |
| Contact (%) |  |  |  |  |  |  |  |  |  |  |  |  |  |
| No | 4772 ( 48.6) | 0 ( 0.0) | 0 ( 0.0) | 0 ( 0.0) | 0 ( 0.0) | 5404 (100.0) | 4758 ( 74.8) | 76 (100.0) | 0 ( 0.0) | 1324 (100.0) | 1462 (100.0) | 0 ( 0.0) | 0 ( 0.0) |
| Yes | 5054 ( 51.4) | 890 (100.0) | 1258 (100.0) | 1414 (100.0) | 148 (100.0) | 0 ( 0.0) | 1595 ( 25.1) | 0 ( 0.0) | 1009 (100.0) | 0 ( 0.0) | 0 ( 0.0) | 1350 (100.0) | 1510 (100.0) |
| Missing | 0 ( 0.0) | 0 ( 0.0) | 0 ( 0.0) | 0 ( 0.0) | 0 ( 0.0) | 0 ( 0.0) | 10 ( 0.2) | 0 ( 0.0) | 0 ( 0.0) | 0 ( 0.0) | 0 ( 0.0) | 0 ( 0.0) | 0 ( 0.0) |
| Previous BCG vaccination (% |  |  |  |  |  |  |  |  |  |  |  |  |  |
| No | 1513 ( 15.4) | 168 ( 18.9) | 470 ( 37.4) | 681 ( 48.2) | 1 ( 0.07) | 3487 ( 64.5) | 56 ( 0.9) | 53 ( 69.7) | 240 ( 23.8) | 0 ( 0.0) | 466 ( 31.9) | 0 ( 0.0) | 361 ( 23.9) |
| Yes | 6763 ( 68.8) | 689 ( 77.4) | 787 ( 62.6) | 733 ( 51.8) | 147 ( 99.3) | 1917 ( 35.5) | 5981 ( 94.0) | 23 ( 30.3) | 570 ( 56.5) | 0 ( 0.0) | 613 ( 41.9) | 0 ( 0.0) | 1149 ( 76.1) |
| Missing | 1550 ( 15.8) | 33 ( 3.7) | 1 ( 0.1) | 0 ( 0.0) | 0 (0.0) | 0 ( 0.0) | 326 ( 5.1) | 0 ( 0.0) | 199 ( 19.7) | 1324 (100.0) | 383 ( 26.2) | 1350 (100.0) | 0 ( 0.0) |
| Previous TB (%) |  |  |  |  |  |  |  |  |  |  |  |  |  |
| No | 9290 ( 94.5) | 873 ( 98.1) | 1257 ( 99.9) | 0 ( 0.0) | 0 ( 0.0) | 0 ( 0.0) | 5709 ( 89.7) | 76 (100.0) | 970 ( 96.1) | 744 ( 56.2) | 1351 ( 92.4) | 0 ( 0.0) | 0 ( 0.0) |
| Yes | 354 ( 3.6) | 17 ( 1.9) | 0 ( 0.0) | 0 ( 0.0) | 148 (100.0) | 0 ( 0.0) | 639 ( 10.0) | 0 ( 0.0) | 36 ( 3.6) | 560 ( 42.3) | 101 ( 6.9) | 0 ( 0.0) | 0 ( 0.0) |
| Missing | 182 ( 1.9) | 0 ( 0.0) | 1 ( 0.1) | 1414 (100.0) | 0 ( 0.0) | 5404 (100.0) | 15 ( 0.2) | 0 ( 0.0) | 3 ( 0.3) | 20 ( 1.5) | 10 ( 0.7) | 1350 (100.0) | 1510 (100.0) |
| HIV-positive (%) |  |  |  |  |  |  |  |  |  |  |  |  |  |
| No | 9091 ( 92.5) | 890 (100.0) | 1238 ( 98.4) | 0 ( 0.0) | 148 (100.0) | 0 ( 0.0) | 6362 (100.0) | 76 (100.0) | 946 ( 93.8) | 0 ( 0.0) | 750 ( 51.3) | 967 ( 71.6) | 1510 (100.0) |
| Yes | 55 ( 0.6) | 0 ( 0.0) | 20 ( 1.6) | 0 ( 0.0) | 0 ( 0.0) | 0 ( 0.0) | 1 ( 0.0) | 0 ( 0.0) | 15 ( 1.5) | 1324 (100.0) | 712 ( 48.7) | 370 ( 27.4) | 0 ( 0.0) |
| Missing | 680 ( 6.9) | 0 ( 0.0) | 0 ( 0.0) | 1414 (100.0) | 0 ( 0.0) | 5404 (100.0) | 0 ( 0.0) | 0 ( 0.0) | 48 ( 4.8) | 0 ( 0.0) | 0 ( 0.0) | 13 ( 1.0) | 0 ( 0.0) |
| BMI (mean (SD)) | 25.69 (6.09) | 23.16 (5.54) | 21.74 (3.18) | NA | 17.52 (2.61) | NA | 22.33 (3.84) | NA | 22.82 (5.27) | 27.16 (5.57) | NA | NA | 20.63 (2.90) |
| TB preventive treatment (%) |  |  |  |  |  |  |  |  |  |  |  |  |  |
| No | 9567 ( 97.4) | 737 ( 82.8) | 633 ( 50.3) | 1347 ( 95.3) | 148 (100.0) | 5404 (100.0) | 6363 (100.0) | 76 (100.0) | 547 ( 54.2) | 663 ( 50.1) | 1330 ( 91.0) | 967 ( 71.6) | 1510 (100.0) |
| Yes | 259 ( 2.6) | 136 ( 15.3) | 623 ( 49.5) | 67 ( 4.7) | 0 ( 0.0) | 0 ( 0.0) | 0 ( 0.0) | 0 ( 0.0) | 11 ( 1.1) | 661 ( 49.9) | 126 ( 8.6) | 0 ( 0.0) | 0 ( 0.0) |
| Missing | 0 ( 0.0) | 17 ( 1.9) | 2 ( 0.2) | 0 ( 0.0) | 0 ( 0.0) | 0 ( 0.0) | 0 ( 0.0) | 0 ( 0.0) | 451 ( 44.7) | 0 ( 0.0) | 6 ( 0.4) | 383 ( 28.4) | 0 ( 0.0) |
| Active TB (%) | 96 ( 1.0) | 30 ( 3.4) | 14 ( 1.1) | 19 ( 1.3) | 0 ( 0.0) | 12 ( 0.2) | 87 ( 1.4) | 2 ( 2.6) | 21 ( 2.1) | 90 ( 6.8) | 10 ( 0.7) | 62 ( 4.6) | 75 ( 5.0) |

TB: tuberculosis; SD: standard deviation; IQR: interquartile range; BCG: bacillus Calmette−Guérin: QFT-GIT: QuantiFERON Gold in Tube; BMI: body mass index; TST: tuberculin skin test

## Table A5. Quality assessment of included studies

|  | Representativeness of the exposed cohort | Selection of the non-exposed cohort | Ascertainment of TB infection test result | Assessment for prevalent TB at start of study | Assessment of outcome | | Follow-up duration | Adequacy of follow up | Score (max 8) |
| --- | --- | --- | --- | --- | --- | --- | --- | --- | --- |
|  |  |  |  |  | Blind assessment | Bacteriological confirmation or record linkage |  |  |  |
| Abubakar 2018 | * | * | * | * |  | * | * | * | 7 |
| Acuna-Villaorduna 2018 | * | * | * | * |  | * | * | * | 7 |
| Altet 2015 | * | * | * | * |  | * | * | * | 7 |
| Diel 2011 | * | * | * | * |  | * | * | * | 7 |
| Huerga 2019 | * | * | * | * |  | * | * | * | 7 |
| Lu 2021 | * | * | * | * |  | * | * | * | 7 |
| Mahomed 2011 | * | * | * | * |  | * | * | * | 7 |
| Muñoz 2015 | * | * | * | * |  | * | * | * | 7 |
| Paradkar 2020 | * | * | * | * |  | * | * | * | 7 |
| Rangaka 2014 | * | * | * | * |  | * | * | * | 7 |
| Sester 2014 | * | * | * |  |  | * | * | * | 6 |
| Shanaube 2014 | * | * | * |  |  | * | * | * | 6 |
| Sharma 2017 | * | * | * | * |  | * | * | * | 7 |

TB: tuberculosis

## Figure A1. Cumulative incidence of TB stratified by test results


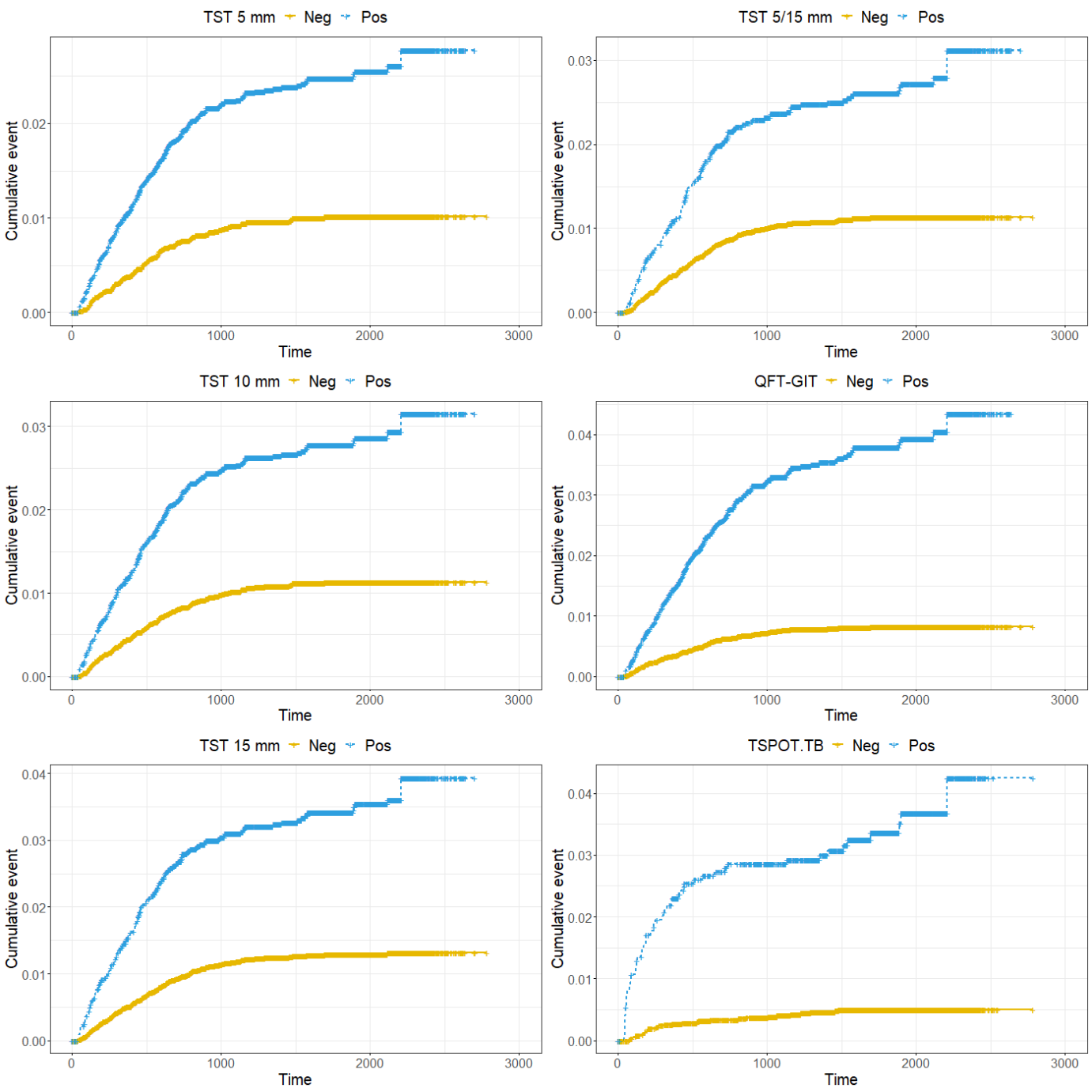


TB: tuberculosis; TST: tuberculin skin test QFT-GIT: QuantiFERON Gold in Tube

The plots were drawn using raw data from 13 studies (3 for TSPOT.TB) combined together.

Test for proportional assumption: p=0.492 for TST ^5 mm^, p=0.476 for TST^10 mm^, p=0.181 for TST^15 mm^, p=0.630 for TST^5/15 mm^, p=0.137 for QFT-GIT, p=0.053 for TSPOT.TB

## Figure A2. Forest plots of the predictive performance of TST vs QFT-GIT for all TB


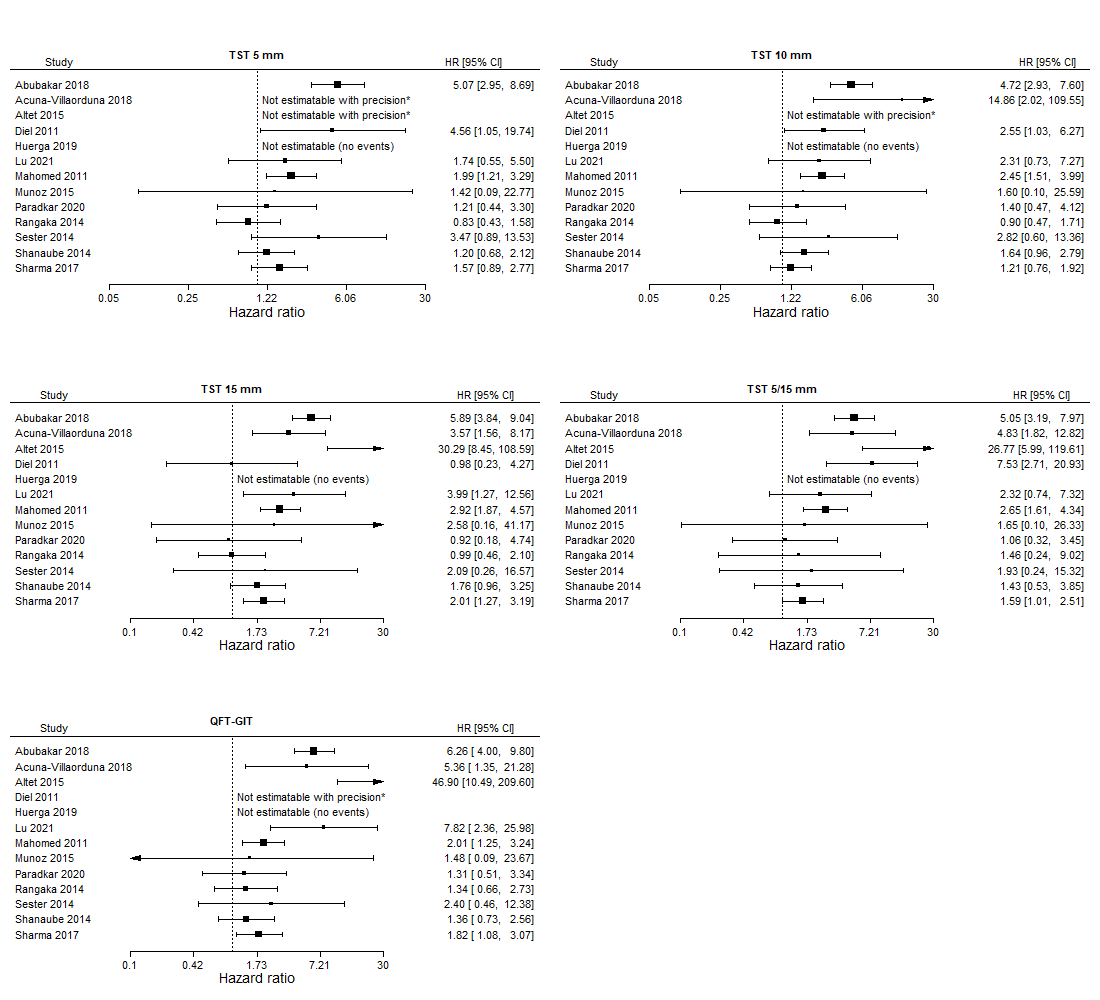


*Extremely large standard errors resulting in 95% confidence intervals ranging from 0 to infinity.

HR indicates the risk of TB in participants with positive test results relative to those with negative results.

TB: tuberculosis; HR: hazard ratio; CI: confidence interval; QFT-GIT: QuantiFERON Gold in Tube; TST: tuberculin skin test

## Figure A3. Predictive performance of TST vs QFT-GIT for all TB using two-stage Poission regression meta-analysis


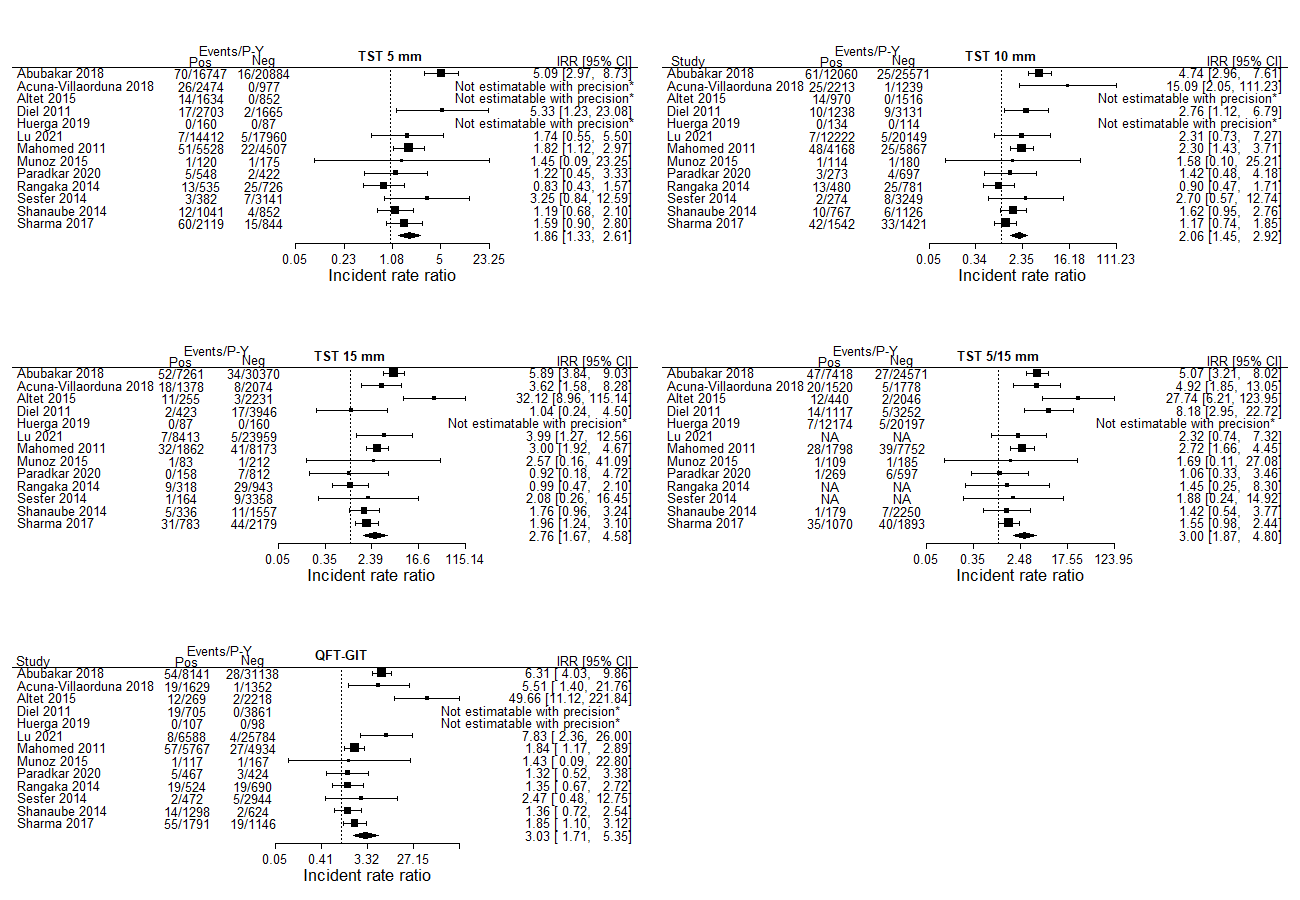


TB: tuberculosis; IRR: incident rate ratio; CI: confidence interval; QFT-GIT: QuantiFERON Gold in Tube; TST: tuberculin skin test; Pos: positive results; Neg: Negative results; P-Y: person-year

IRR indicates the risk of TB in participants with positive test results relative to those with negative results.

The number of events and person-year are based on complete cases, while the estimates are based on multiply imputed datasets. For Huerga 2019, Rangaka 2014, and Sester 2014, bacille Calmette-Guerin vaccination status was not available; hence, data were imputed to estimate the predictive performance using TST^5/15 mm^.

## Table A6. Comparison of the predictive performance of TST vs IGRA for all TB

| TST cut-off | **The ratio of two odds ratios (95% CI)** | | |
| --- | --- | --- | --- |
|  | vs QFT-GIT (13 studies) | vs QFT-GIT  (12 studies, excluding Diel 2011) | vs TSPOT.TB (3 studies) |
| TST 5 mm | 1.18 (0.83-1.67), p= 0.36 | 0.93 (0.64-1.34), p= 0.69 | 0.81 (0.36-1.78), p= 0.59 |
| TST 10 mm | 1.46 (1.01-2.12), p= 0.045 | 1.35 (0.94-1.95), p= 0.11 | 1.6 (0.79-3.26), p= 0.19 |
| TST 15 mm | 1.48 (1.03-2.13), p= 0.035 | 1.23 (0.84-1.8), p= 0.28 | 1.53 (0.62-3.81), p= 0.35 |
| TST 5/15 mm | 1.14 (0.72-1.81), p= 0.58 | 0.97 (0.62-1.53), p= 0.9 | 0.92 (0.4-2.13), p= 0.84 |

Values indicate the ratio of two odds ratios for TST and IGRA, respectively, for positive test results in participants who developed active TB compared with those who did not. A value above 1 indicates a positive result on either QFT-GIT or TSPOT.TB predicts TB development better than a positive result on TST with the corresponding cut-off value.

TB: tuberculosis; CI: confidence interval; QFT-GIT: QuantiFERON Gold in Tube; TST: tuberculin skin test

## Figure A4. Predictive performance of TST vs TSPOT.TB for all TB


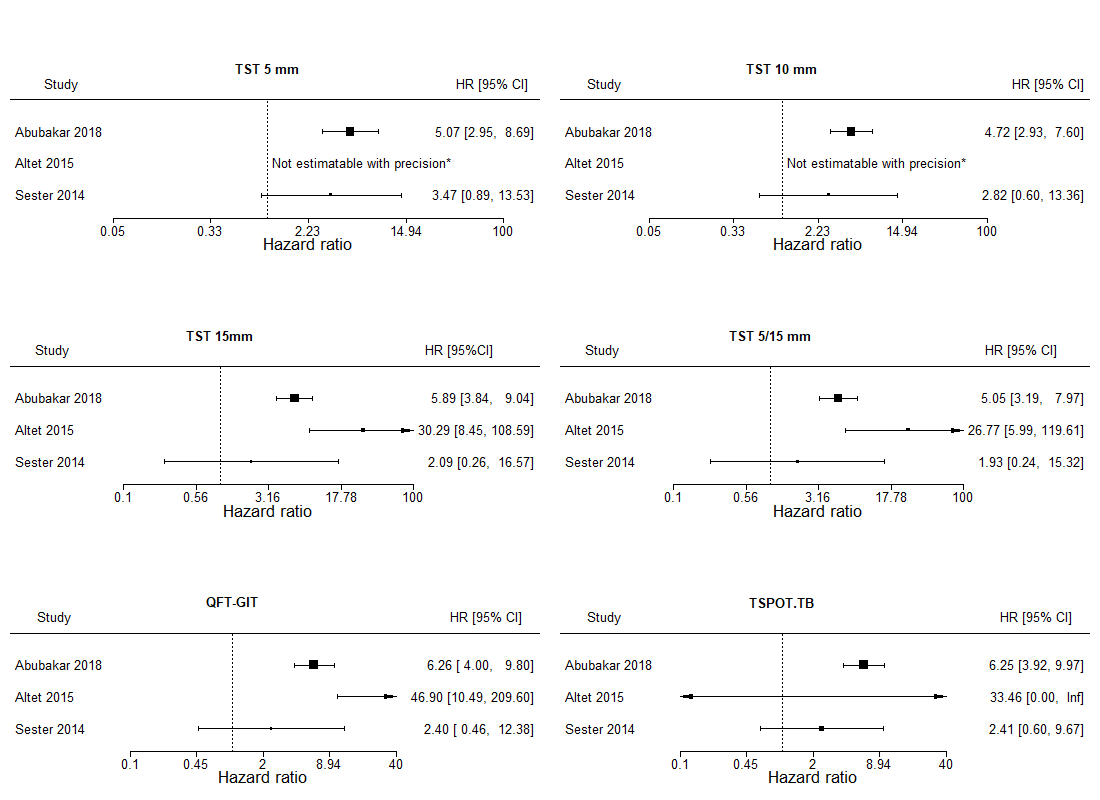


TB: tuberculosis; HR: hazard ratio; CI: confidence interval; QFT-GIT: QuantiFERON Gold in Tube; TST: tuberculin skin test

HR indicates the risk of TB in participants with positive test results relative to those with negative results.

## Figure A5. Predictive performance of TST vs TSPOT.TB for all TB using two-stage Poission regression meta-analysis


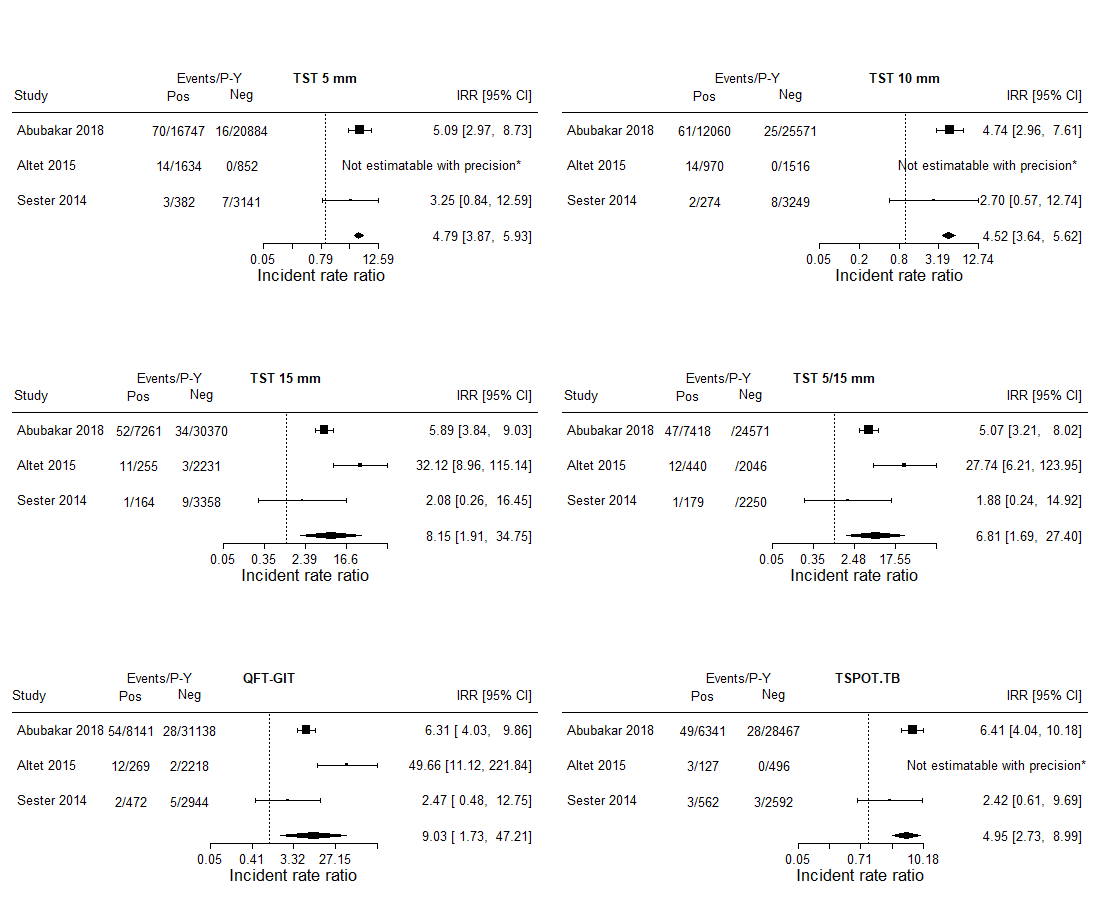


TB: tuberculosis; IRR: incident rate ratio; CI: confidence interval; QFT-GIT: QuantiFERON Gold in Tube; TST: tuberculin skin test; Pos: positive results; Neg: Negative results; P-Y: person-year

The number of events and person-year are based on complete cases, while the estimates are based on multiply imputed datasets.

IRR indicates the risk of TB in participants with positive test results relative to those with negative results.

## Table A7. Pooled estimates of the predictive performance of TST vs QFT-GIT-sensitivity analysis

|  | Only microbiologically confirmed TB | | Exclude TB diagnosed within < 14 days as prevalent TB | | Exclude TB diagnosed within < 180 days as prevalent TB | |
| --- | --- | --- | --- | --- | --- | --- |
| Test | HR (95% CI) | I^2^ (95% CI) | HR (95% CI) | I^2^ (95% CI) | HR (95% CI) | I^2^ (95% CI) |
| TST5 mm | 2.34 (1.4-3.9) | 15.7 (0-55.4) | 2.48 (1.52-4.06) | 66.7 (38.8-81.9) | 2.11 (1.31-3.41) | 66.7 (38.8-81.9) |
| TST10 mm | 2.52 (1.59-3.98) | 21.4 (0-59.6) | 2.69 (1.66-4.37) | 71.7 (49.3-84.2) | 2.47 (1.54-3.97) | 71.7 (49.3-84.2) |
| TST15 mm | 2.96 (1.89-4.64) | 36.5 (0-67.9) | 2.72 (1.6-4.63) | 78.7 (63.4-87.6) | 2.7 (1.56-4.68) | 78.7 (63.4-87.6) |
| TST5/15 mm | 2.9 (1.59-5.31) | 28.8 (0-64) | 2.93 (1.72-5.01) | 69.4 (44.6-83.2) | 2.9 (1.57-5.35) | 69.4 (44.6-83.2) |
| QFT-GIT | 4.45 (2.16-9.16) | 46.7 (0-72.7) | 4.25 (2.04-8.84) | 76.8 (59.6-86.7) | 4.41 (2.09-9.29) | 76.8 (59.6-86.7) |

TB: tuberculosis; HR: hazard ratio; CI: confidence interval; QFT-GIT: QuantiFERON Gold in Tube; TST: tuberculin skin test

HR indicates the risk of TB in participants with positive test results relative to those with negative results.

## Table A8. Pooled estimates of the predictive performance of TST vs TSPOT.TB-sensitivity analysis

|  | Only microbiologically confirmed TB | | Exclude TB diagnosed within < 14 days as prevalent TB | | Exclude TB diagnosed within < 180 days as prevalent TB | |
| --- | --- | --- | --- | --- | --- | --- |
| Test | HR (95% CI) | I^2^ (95% CI) | HR (95% CI) | I^2^ (95% CI) | HR (95% CI) | I^2^ (95% CI) |
| TST5 mm | 5.74 (3.01-10.95) | 0 (0-89.6) | 6.11 (3.84-9.73) | 0 (0-89.6) | 4.61 (2.57-8.28) | 0 (0-89.6) |
| TST10 mm | 4.97 (2.87-8.6) | 0 (0-89.6) | 6.17 (4.09-9.31) | 0 (0-89.6) | 4.52 (2.72-7.49) | 0 (0-89.6) |
| TST15 mm | 6.85 (2.85-16.44) | 41.8 (0-82.4) | 7.71 (5.37-11.06) | 73.4 (10.9-92.1) | 6.27 (1.18-33.3) | 73.4 (10.9-92.1) |
| TST5/15 mm | 5.59 (2.31-13.52) | 31.8 (0-92.9) | 6.17 (4.2-9.05) | 60 (0-88.6) | 5.97 (1.14-31.17) | 60 (0-88.6) |
| QFT-GIT | 9.18 (2.52-33.35) | 71.2 (2.3-91.5) | 9.08 (3.63-22.7) | 67.7 (0-90.7) | 9.32 (4.13-21.05) | 67.7 (0-90.7) |
| TSPOT.TB | 6.35 (3.37-11.99) | 0 (0-89.6) | 7.46 (4.14-13.43) | 0.8 (0-89.7) | 4.89 (2.09-11.45) | 0.8 (0-89.7) |

TB: tuberculosis; HR: hazard ratio; CI: confidence interval; QFT-GIT: QuantiFERON Gold in Tube; TST: tuberculin skin test

HR indicates the risk of TB in participants with positive test results relative to those with negative results.

## Figure A6. Funnel plots and p values for Egger’s test


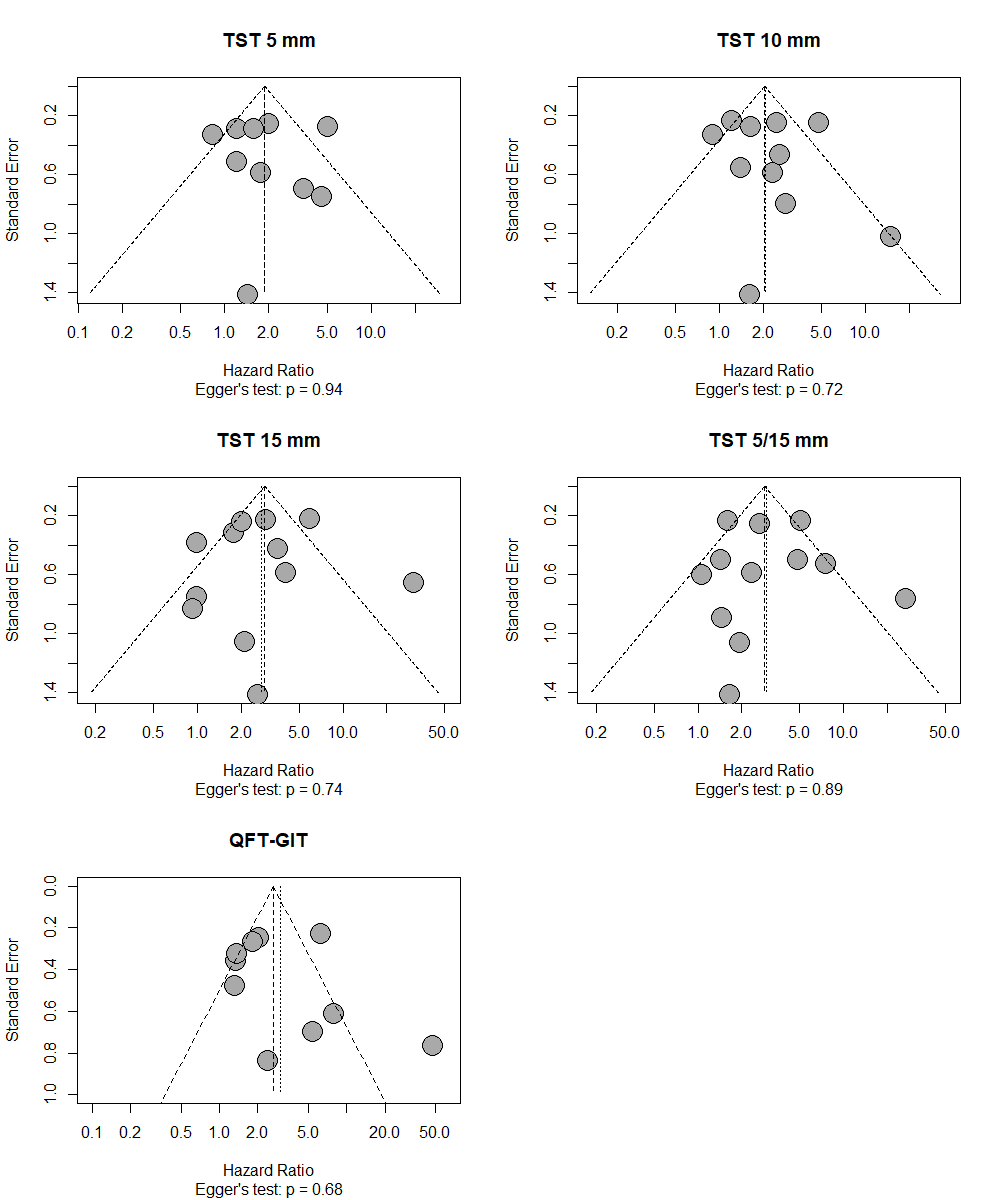


QFT-GIT: QuantiFERON Gold in Tube; TST: tuberculin skin test

No plot is shown for TSPOT.TB because there were only three studies.

## Table A9. The differences in the predictive performance of TST and QFT-GIT for all TB- the ratio of hazard ratios

| Variable | The ratio of hazard ratios (95% CI) and p-value for within-study interaction | | | | |
| --- | --- | --- | --- | --- | --- |
|  | TST 5 mm | TST 10 mm | TST 15 mm | TST 5/15 mm | QFT-GIT |
| Age per 10 -year increase | 0.97 (0.96-0.99), p= 0.003 | 0.98 (0.96-0.99), p= 0.0032 | 0.97 (0.96-0.99), p= 0.0017 | 0.98 (0.96-1), p= 0.026 | 1 (0.98-1.01), p= 0.58 |
| Adults vs Children and adolescents | 0.46 (0.21-1), p= 0.049 | 0.44 (0.23-0.83), p= 0.012 | 0.33 (0.17-0.62), p= 0.00067 | 0.37 (0.2-0.7), p= 0.0019 | 0.95 (0.47-1.94), p= 0.89 |
| Contact vs non-Contact | 0.77 (0.36-1.62), p= 0.48 | 0.73 (0.38-1.42), p= 0.35 | 0.59 (0.31-1.11), p= 0.1 | 0.7 (0.36-1.36), p= 0.29 | 0.77 (0.4-1.47), p= 0.42 |
| TB incidence  100 ≥ vs <100 | 0.28 (0.16-0.47), p= <0.0001 | 0.31 (0.18-0.55), p= <0.0001 | 0.37 (0.17-0.82), p= 0.014 | 0.31 (0.16-0.63), p= 0.0013 | 0.15 (0.06-0.34), p= <0.0001 |
| BMI per 1 kg/m^2^ increase | 0.99 (0.94-1.05), p= 0.71 | 0.98 (0.92-1.03), p= 0.44 | 1 (0.95-1.06), p= 0.94 | 0.99 (0.93-1.06), p= 0.86 | 0.99 (0.93-1.05), p= 0.68 |
| Underweight vs normal weight | 0.82 (0.41-1.63), p= 0.57 | 0.86 (0.44-1.69), p= 0.66 | 0.71 (0.36-1.43), p= 0.34 | 0.94 (0.42-2.08), p= 0.87 | 0.92 (0.43-2.01), p= 0.84 |
| Overweight vs normal weight | 0.81 (0.45-1.45), p= 0.47 | 0.69 (0.37-1.26), p= 0.22 | 0.8 (0.44-1.45), p= 0.46 | 0.84 (0.45-1.58), p= 0.58 | 0.86 (0.47-1.57), p= 0.61 |
| HIV-positive vs HIV-negative | 0.7 (0.26-1.88), p= 0.48 | 0.83 (0.31-2.22), p= 0.71 | 0.83 (0.29-2.43), p= 0.74 | 0.75 (0.22-2.56), p= 0.65 | 0.51 (0.17-1.54), p= 0.23 |

The values indicate the changes (ratios) in hazard ratios by sub-group or changes in the covariate. They were estimated by including interaction terms by separating within-study and between-study interactions.

Disaggregation of individuals into children and adolescents would have been preferable, but a low sample size of individuals < 18 years old precluded this.

TB: tuberculosis; CI: confidence interval; QFT-GIT: QuantiFERON Gold in Tube; TST: tuberculin skin test

## Figure A7. Predictive performance of TST vs QFT-GIT for all TB by TB incidence in study countries, excluding HIV-positive participants


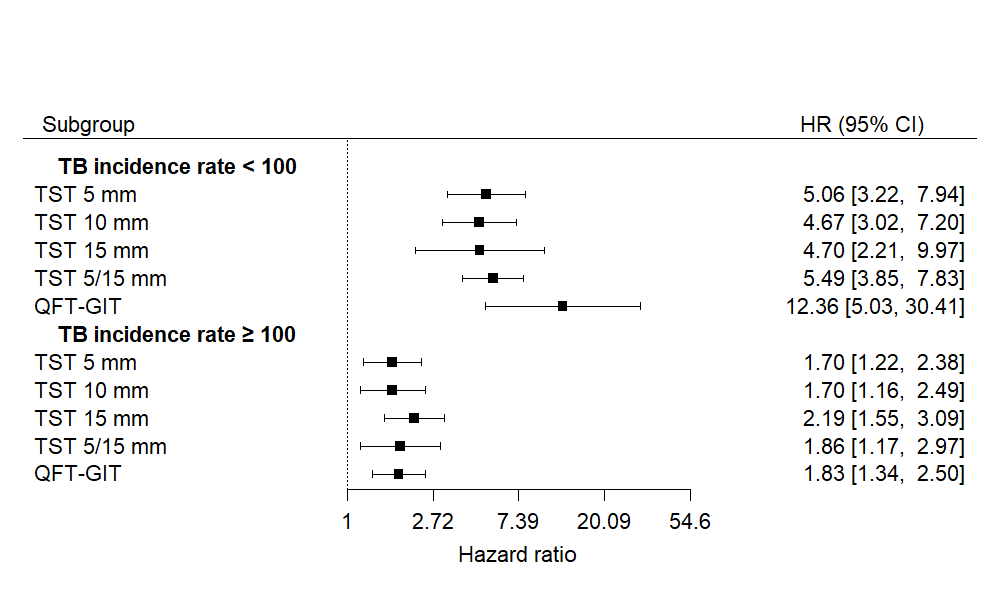


HR: hazard ratio; CI: confidence interval; QFT-GIT: QuantiFERON Gold in Tube; TST: tuberculin skin test

HR indicates the risk of TB in participants with positive test results relative to those with negative results.

## Figure A8. Sensitivity and specificity for predicting the development of active TB over two years


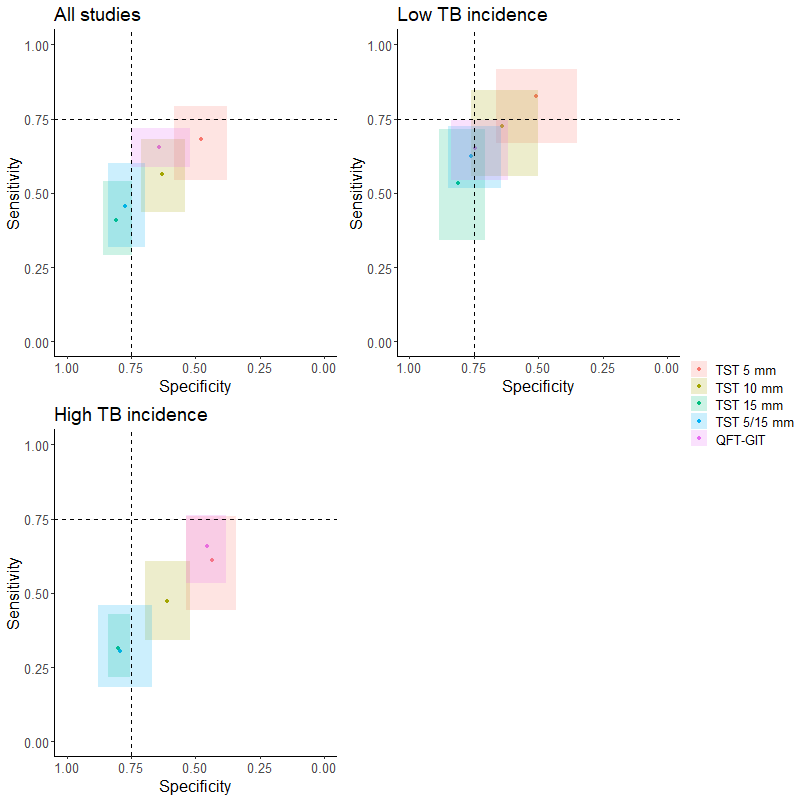


Note: WHO minimum targets are ≥ 75% for both sensitivity and specificity.

High TB incidence: TB incidence rate ≥ 100/100,000 population

Low TB incidence: TB incidence rate < 100/100,000 population

QFT-GIT: QuantiFERON Gold in Tube; TST: tuberculin skin test

## Figure A9. Sensitivity and specificity for predicting the development of active TB over two years- studies reporting TSPOT.TB


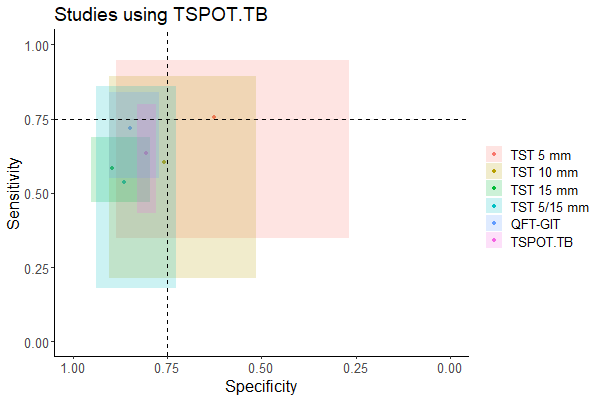


Note: World Health Organization minimum targets are ≥ 75% for both sensitivity and specificity.

QFT-GIT: QuantiFERON Gold in Tube; TST: tuberculin skin test

## Table A10. Sensitivity and specificity for predicting the development of active TB over two years

|  | All studies | | Low TB incidence | | High TB incidence | | TSPOT.TB studies | |
| --- | --- | --- | --- | --- | --- | --- | --- | --- |
| Test | Sensitivity (95% CI) | Specificity (95% CI) | Sensitivity (95% CI) | Specificity (95% CI) | Sensitivity (95% CI) | Specificity (95% CI) | Sensitivity (95% CI) | Specificity (95% CI) |
| TST 5 mm | 0.68 (0.54-0.79) | 0.48 (0.38-0.58) | 0.83 (0.67-0.92) | 0.51 (0.35-0.66) | 0.61 (0.44-0.76) | 0.44 (0.34-0.54) | 0.76 (0.35-0.95) | 0.63 (0.27-0.89) |
| TST 10 mm | 0.56 (0.44-0.68) | 0.63 (0.54-0.71) | 0.72 (0.56-0.85) | 0.64 (0.5-0.76) | 0.47 (0.34-0.61) | 0.61 (0.52-0.69) | 0.6 (0.22-0.89) | 0.76 (0.51-0.9) |
| TST 15 mm | 0.41 (0.29-0.54) | 0.81 (0.74-0.86) | 0.53 (0.34-0.72) | 0.81 (0.71-0.89) | 0.31 (0.22-0.43) | 0.8 (0.75-0.84) | 0.58 (0.47-0.69) | 0.9 (0.79-0.95) |
| TST 5/15 mm | 0.45 (0.32-0.6) | 0.77 (0.69-0.84) | 0.63 (0.52-0.72) | 0.76 (0.65-0.85) | 0.3 (0.18-0.46) | 0.79 (0.67-0.88) | 0.54 (0.18-0.86) | 0.86 (0.73-0.94) |
| QFT-GIT | 0.66 (0.59-0.72) | 0.64 (0.52-0.75) | 0.65 (0.54-0.75) | 0.74 (0.62-0.84) | 0.66 (0.53-0.76) | 0.46 (0.38-0.54) | 0.72 (0.55-0.84) | 0.85 (0.77-0.9) |
| TSPOT.TB | - | - | - | - | - | - | 0.63 (0.43-0.8) | 0.81 (0.78-0.83) |

CI: confidence interval; QFT-GIT: QuantiFERON Gold in Tube; TST: tuberculin skin test

## Table A11. Positive and negative predictive value in a hypothetical population (n = 10,000)

| Test | Sensitivity | Specificity | TP | FP | FN | TN | PPV (%) | NPV (%) | PPV/(100-NPV) |
| --- | --- | --- | --- | --- | --- | --- | --- | --- | --- |
| Countries with TB incidence rate < 100 per 100,000: pre-test probability of incident TB over 2 years = 1% | | | | | | | | | |
| TST 5 mm | 0.83 | 0.51 | 83 | 4851 | 17 | 5049 | 1.68 | 99.66 | 4.94 |
| TST 10 mm | 0.72 | 0.64 | 72 | 3564 | 28 | 6336 | 1.98 | 99.56 | 4.5 |
| TST 15 mm | 0.53 | 0.81 | 53 | 1881 | 47 | 8019 | 2.74 | 99.42 | 4.72 |
| TST 5/15 mm | 0.63 | 0.76 | 63 | 2376 | 37 | 7524 | 2.58 | 99.51 | 5.27 |
| QFT-GIT | 0.65 | 0.74 | 65 | 2574 | 35 | 7326 | 2.46 | 99.52 | 5.12 |
| Countries with TB incidence rate ≥ 100 per 100,000: pre-test probability of incident TB over 2 years = 5% | | | | | | | | | |
| TST 5 mm | 0.61 | 0.44 | 305 | 5320 | 195 | 4180 | 5.42 | 95.54 | 1.22 |
| TST 10 mm | 0.47 | 0.61 | 235 | 3705 | 265 | 5795 | 5.96 | 95.63 | 1.36 |
| TST 15 mm | 0.31 | 0.8 | 155 | 1900 | 345 | 7600 | 7.54 | 95.66 | 1.74 |
| TST 5/15 mm | 0.3 | 0.79 | 150 | 1995 | 350 | 7505 | 6.99 | 95.54 | 1.57 |
| QFT-GIT | 0.66 | 0.46 | 330 | 5130 | 170 | 4370 | 6.04 | 96.26 | 1.61 |

QFT-GIT: QuantiFERON Gold in Tube; TST: tuberculin skin test; TP: true positive; FP: false positive; FN: false negative; TN: true negative; PPV: positive predictive value; NPV: negative predictive value

## Figure A10. Predictive performance of TST vs QFT-GIT for all TB, Adults vs Children and adolescents


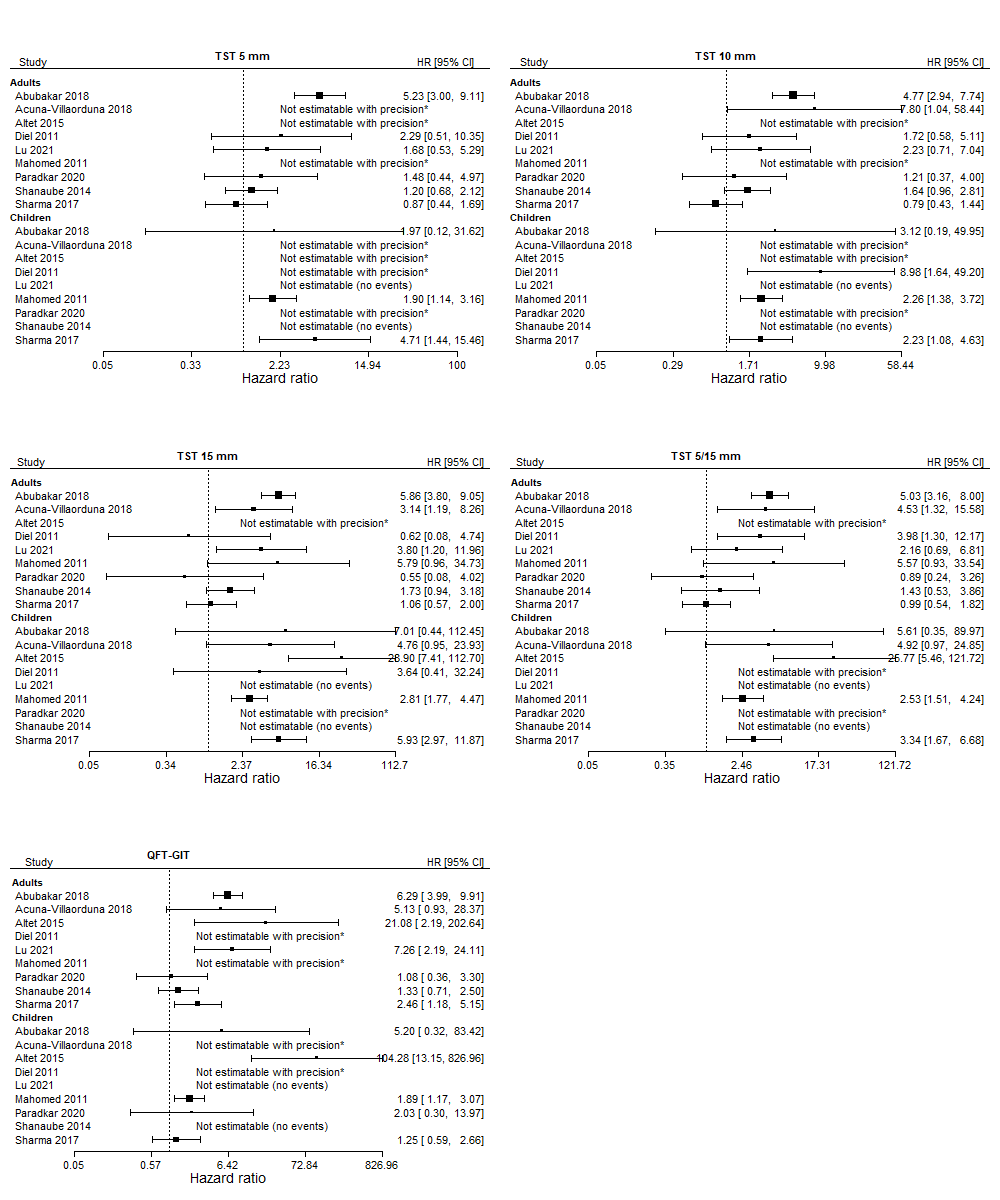


*Extremely large standard errors resulting in 95% confidence intervals ranging from 0 to infinity.

Huerga 2019, Munos 2015, Rangaka 2014, and Sester 2014 included only adults or children only and did not allow within-study comparisons.

HR indicates the risk of TB in participants with positive test results relative to those with negative results.

HR: hazard ratio; CI: confidence interval; QFT-GIT: QuantiFERON Gold in Tube; TST: tuberculin skin test

## Figure A11. Predictive performance of TST vs QFT-GIT for all TB by contact history


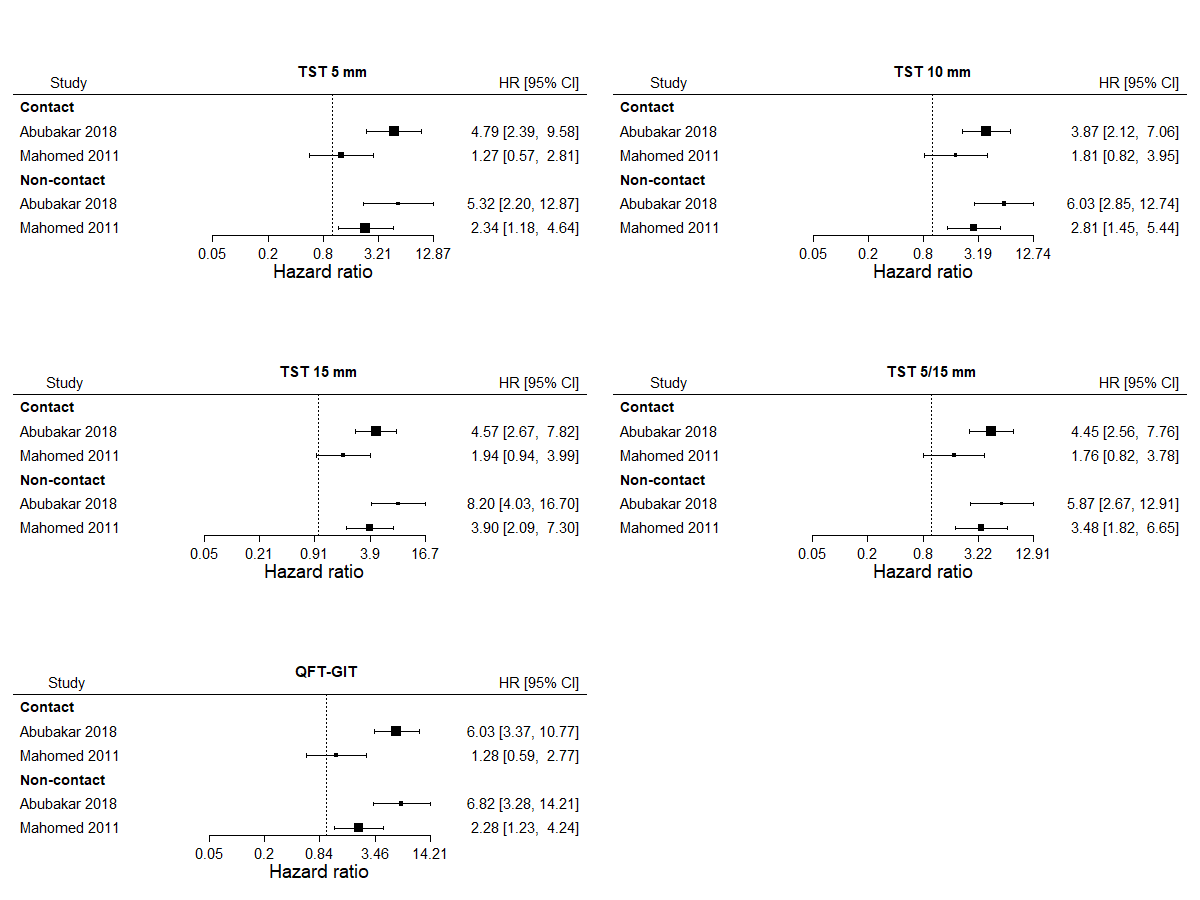


HR: hazard ratio; CI: confidence interval; QFT-GIT: QuantiFERON Gold in Tube; TST: tuberculin skin test

For contact history, only two studies included contacts and non-contacts, resulting in imprecise estimates.^14,18^

HR indicates the risk of TB in participants with positive test results relative to those with negative results.

## Table A12. The differences in the predictive performance in studies with TSPOT.TB data- the ratio of hazard ratios

| Variable | The ratio of hazard ratios (95% CI) and p-value for within-study interaction | | | | |  |
| --- | --- | --- | --- | --- | --- | --- |
|  | TST 5 mm | TST 10 mm | TST15 mm | TST 5/15 mm | QFT-GIT | TSPOT.TB |
| Age per 10 -year increase | 0.97 (0.93-1), p= 0.03 | 0.97 (0.94-1), p= 0.029 | 0.99 (0.96-1.02), p= 0.4 | 0.98 (0.95-1.02), p= 0.31 | 0.98 (0.95-1.02), p= 0.33 | 0.97 (0.94-1.01), p= 0.11 |
| Adults vs Children and adolescents | 1.67 (0.15-18.37), p= 0.67 | 0.81 (0.07-8.86), p= 0.86 | 1.17 (0.2-7.06), p= 0.86 | 0.94 (0.14-6.15), p= 0.95 | 0.43 (0.06-3.07), p= 0.4 | 0.89 (0.1-7.68), p= 0.91 |
| Contact vs non-Contact | 0.86 (0.28-2.62), p= 0.79 | 0.65 (0.24-1.74), p= 0.39 | 0.55 (0.23-1.33), p= 0.19 | 0.69 (0.26-1.81), p= 0.45 | 0.85 (0.33-2.21), p= 0.74 | 0.68 (0.27-1.68), p= 0.4 |
| BMI | 0.95 (0.86-1.06), p= 0.41 | 0.97 (0.87-1.08), p= 0.57 | 1 (0.89-1.12), p= 0.96 | 1.01 (0.92-1.12), p= 0.83 | 0.97 (0.88-1.08), p= 0.63 | 0.97 (0.88-1.07), p= 0.49 |
| Underweight vs normal weight | 2.64 (0.14-48.75), p= 0.51 | 1.78 (0.21-14.93), p= 0.59 | 0.89 (0.17-4.82), p= 0.9 | 2.66 (0.35-20.25), p= 0.34 | 1.78 (0.25-12.83), p= 0.57 | 1.57 (0.19-12.87), p= 0.67 |
| Overweight vs normal weight | 0.76 (0.25-2.31), p= 0.62 | 0.74 (0.26-2.1), p= 0.58 | 0.66 (0.26-1.68), p= 0.38 | 0.95 (0.35-2.59), p= 0.92 | 0.87 (0.29-2.62), p= 0.8 | 0.97 (0.35-2.67), p= 0.95 |
| HIV-positive vs HIV-negative | 6.75 (0.09-490.51), p= 0.38 | 5.13 (0.09-305.52), p= 0.43 | 1.82 (0.04-81.23), p= 0.76 | 1.45 (0.03-61), p= 0.85 | 0.38 (0-46.71), p= 0.69 | 2.6 (0.08-85.78), p= 0.59 |

The values indicate the changes (ratios) in hazard ratios by sub-group or changes in the covariate. They were estimated by including interaction terms by separating within-study and between-study interactions.

BMI: body mass index; CI: confidence interval; QFT-GIT: QuantiFERON Gold in Tube; TST: tuberculin skin test

## Figure A12. Studies that did not provide IPD

**
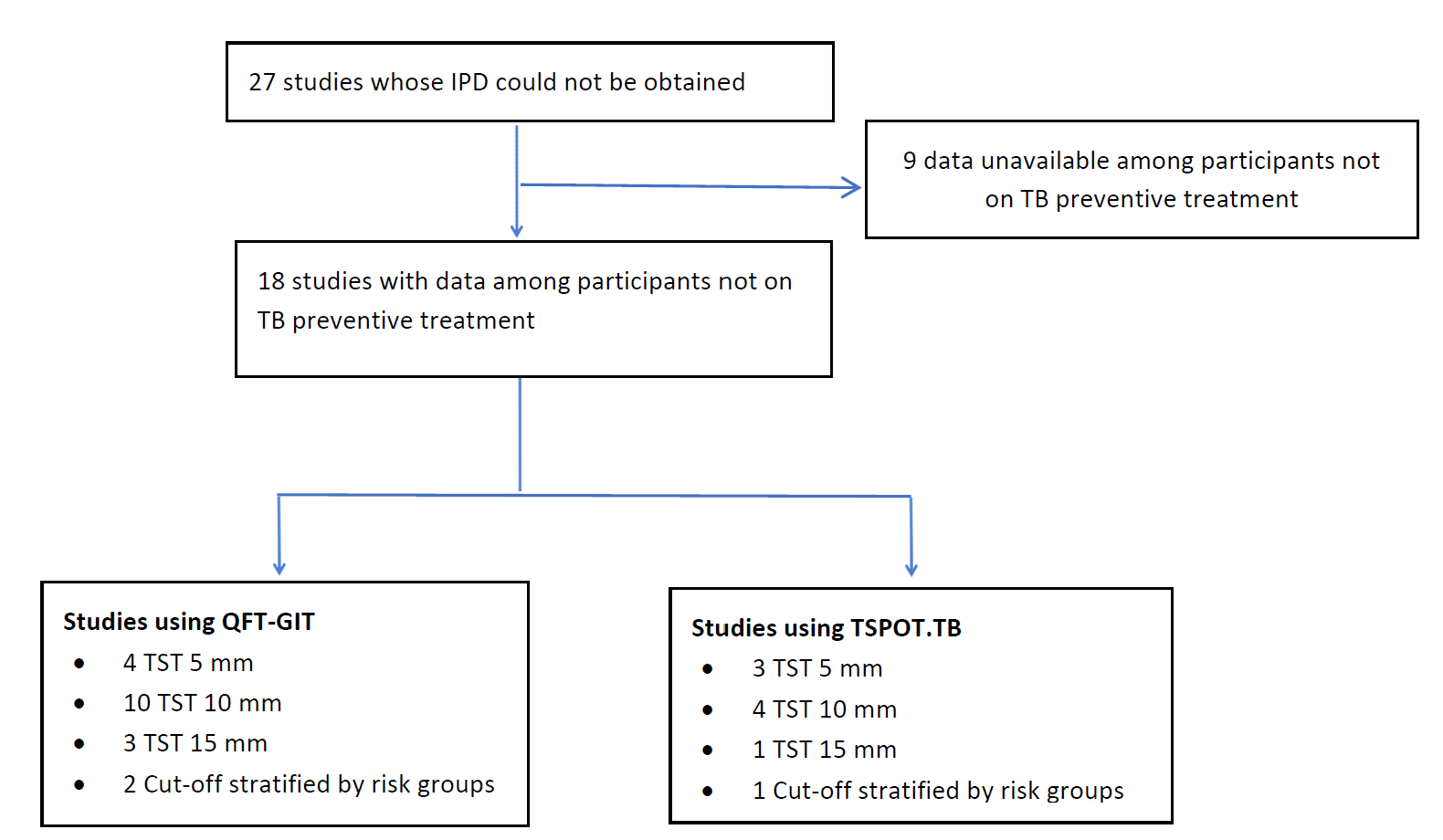
**

**Note:** Four studies used both QFT-GIT and TSPOT.TB

QFT-GIT: QuantiFERON Gold in Tube; TST: tuberculin skin test

## Figure A13. Meta-analysis of the predictive performance of TST vs QFT-GIT for all TB stratified by the availability of IPD


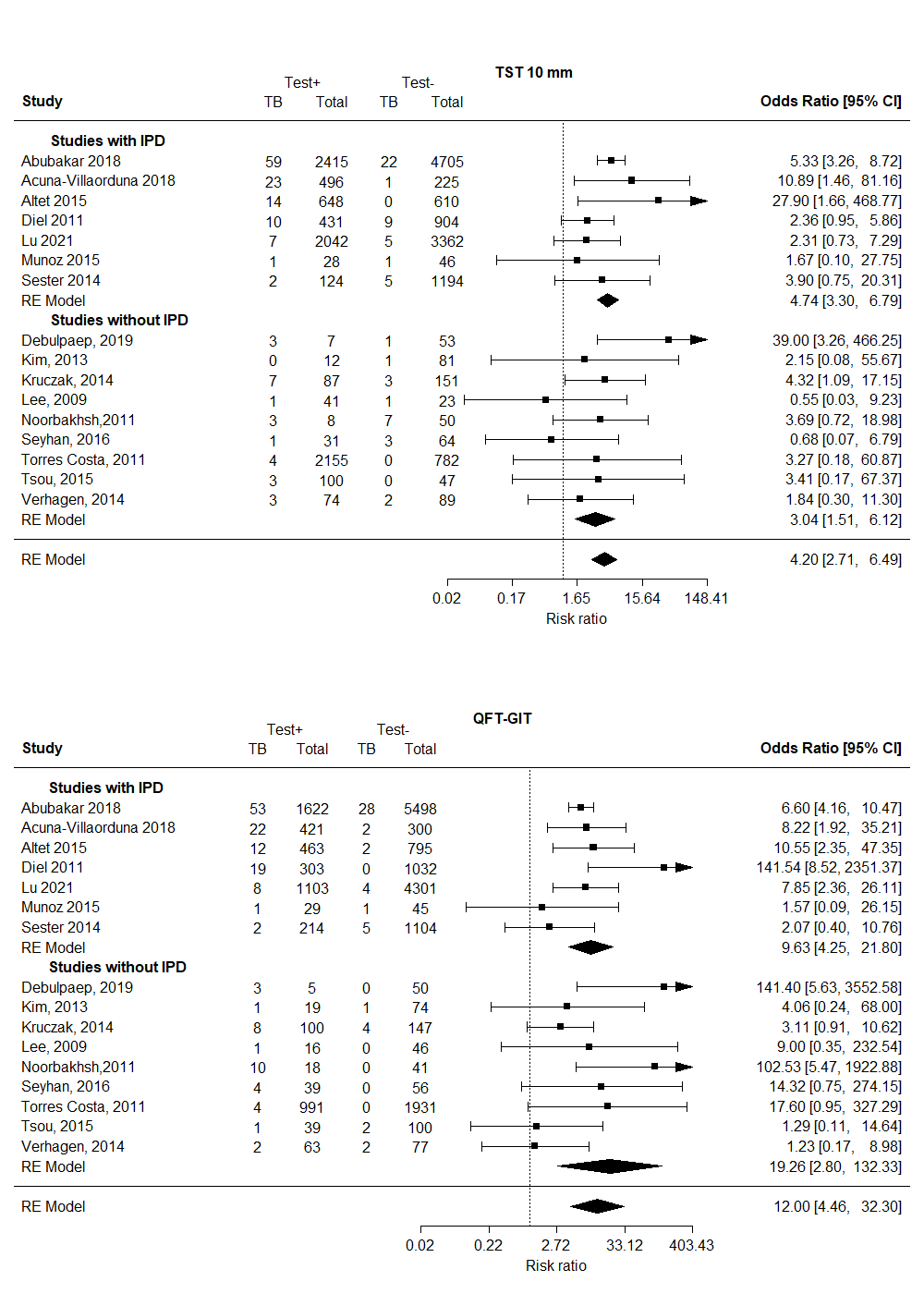


TB: tuberculosis; QFT-GIT: QuantiFERON Gold in Tube; TST: tuberculin skin test; RE: random effect; CI: confidence interval

We meta-analysed studies that used TST^10 mm^ and QFT-GIT since that was the largest set of studies that could be included.

Huerga, 2019 and Ringshausen, 2010 were not included because of no events.
